# Revealing Fibrosis Genes as Biomarkers of Ulcerative Colitis: A Bioinformatics Study Based on ScRNA and Bulk RNA Datasets

**DOI:** 10.2174/0118715303332155240912050838

**Published:** 2024-10-18

**Authors:** Yandong Wang, Li Liu, Weihao Wang

**Affiliations:** 1 College of Nursing and Rehabilitation, North China University of Science and Technology, Tangshan, 063000, China;; 2 Department of Intensive Care Medicine, North China University of Science and Technology Affiliated Hospital,Tangshan, 063000, China;; 3 School of Chemical and Biological Engineering, Yichun University, Yichun, 336000, Jiangxi, China

**Keywords:** Ulcerative colitis, single-cell sequencing, fibrosis, machine learning, bioinformatics, TNFa inhibitor

## Abstract

**Objective:**

This study aimed to uncover biomarkers associated with fibroblasts to diagnose ulcerative colitis (UC) and predict sensitivity to TNFα inhibitors.

**Methods:**

We identified fibrosis-related genes by analyzing eight bulk RNA and one single-cell RNA sequencing dataset from UC patients. Three machine learning algorithms were employed to identify common significant genes. We utilized five machine learning models, namely Random Forest (RF), Support Vector Machine (SVM), Xgboost, Multilayer Perceptron (MLP), and Logistic Regression, to develop diagnostic models for UC. Following hyperparameter tweaking using grid search, we evaluated Matthew’s Correlation Coefficient (MCC) of each model on the validation set. Finally, we identified five hub genes in UC patients and evaluated their response to infliximab or golimumab.

**Results:**

We identified 23 genes associated with fibroblasts. Further analysis using three ML models revealed BIRC3, IFITM2, ANXA1, ISG20, and MSN as critical fibroblast genes. Following hyperparameter adjustment, the SVM model exhibited the most favorable characteristics in the validation set, achieving an MCC of 0.7. ANXA1 contributed the most to the model that predicts UC. The optimal model was implemented on the website. Among UC patients receiving TNFα inhibitor treatment, the ineffective group showed considerably increased expression of the five critical genes than the responsive group.

**Conclusion:**

BIRC3, IFITM2, ANXA1, ISG20, and MSN may serve as potential diagnostic biomarkers in UC. Through the interaction between characteristic biomarkers and immune infiltrating cells, the immune response mediated by these characteristic biomarkers plays a crucial role in the occurrence and development of UC.

## 1 INTRODUCTION

Ulcerative colitis (UC) is an autoimmune disease characterized by abdominal pain and diarrhea. Its global occurrence has been increasing, particularly in developing nations. UC can be protracted and challenging to recover from because of factors, such as environmental influences, imbalances in the intestinal flora, and disruptions in immunological homeostasis. This leads to increased treatment expenses and significant psychological stress for patients with UC [1, 2]. Determining the root cause and devising strategies for the effective diagnosis and treatment of UC are crucial.

Fibrosis is the abnormal accumulation of extracellular matrix components due to the excessive proliferation of myofibroblasts. Persistent inflammation leads to excessive wound repair and aberrant healing [3, 4]. Intestinal fibrosis is a remarkable characteristic of UC [5]. Previous studies have reported the occurrence of extracellular matrix deposition in the intestinal tissue of adults with UC. This is intimately linked to the occurrence of intestinal stenosis and abnormal bowel function in patients with UC [6]. Similarly, children with UC showed the presence of submucosal fibrosis in the colon and thickening of the mucosal muscle layer [7]. This study examines the traditional belief that chronic inflammation causes fibrosis. Intestinal tissue fibrosis develops early in UC. Hence, targeting fibrosis-related factors is crucial for diagnosing UC and developing viable treatments.

Given the significant role of fibrosis in UC, understanding the mechanisms of fibrosis-related genes is crucial in deducing UC pathophysiology. Therefore, in this study, we analyzed single-cell RNA and bulk RNA datasets associated with UC to identify differentially expressed genes related to fibrosis. We then used machine learning to identify potential hub genes, develop diagnostic models, and verify their accuracy using external data. We then used the main genes to evaluate treatment efficacy in UC patients administered TNFα inhibitors.

## 2 METHODS

### 2.1 Bulk-RNA Datasets Processing

After setting exclusion criteria (<18 years or patients in recovery) and inclusion criteria (inflammation phase), eight bulk-RNA datasets, namely, GSE107499, GSE179285, GSE36807, GSE38713, GSE53306, GSE65114, GSE75214 and GSE87466 [8, 9], comprising data from 162 normal and 275 UC samples, were obtained from the Gene Expression Omnibus (GEO) database. The R packages “limma” and “RobustRankAggreg” were used to identify differentially expressed genes (DEGs, FDR< 0.01 and |log2FC| > 1) related to UC. As shown in Table **S1**, we obtained 477 DEGs associated with UC (up-regulated:302, down-regulated: 175). The top 10 up-regulated and down-regulated genes were visualised (Fig. **S1A**). Subsequently, the R package “sva” was used to integrate the eight datasets and mitigate the batch effects, and the results were visualized by the R package “ggplot2” (Fig. **S1B**). The degree of fibroblast infiltration between UC and normal tissues was compared by importing the combined datasets into Sangerbox, an online bioinformatics analysis platform [10], by utilizing four methods: Xcell, ESTIMATE, MCPcounter, and EPIC. The fibroblast scores of the two groups were compared using the Wilcoxon test (*p*<0.05). The results showed that patients in the UC group exhibited increased fibroblast infiltration (Figs. **S1C**-**S1F**).

### 2.2 Single-cell Sequencing Data Analysis

First, a ScRNA dataset (GSE214695) [11] was comprised of 6 cases each of UC in the inflammatory phase and normal tissues. The R package “Seurat” was used to analyze the data. The data were subjected to quality control based on the following filtering conditions: nCount_RNA≥1000,200 ≤nFeature_RNA≤10000, percent.mt≤15, and percent.rb≤20. Ineligible cells were accordingly filtered out. As shown in Figs. (**S2A** and **S2B**), after mitigating the batch effect and obtaining the optimal PC=17, we performed dimensionality reduction and clustering on the data (Fig. **S2C**). Cell types were automatically annotated using Single R, as shown in Fig. (**S2D**), including fibroblasts, B cells, monocytes, M1/M2 macrophages, epithelial cells, endothelial cells, and CD4^+^ memory T cells. The proportion of fibroblasts was significantly increased in UC tissues (Fig. **S2E**) [12].

Furthermore, we extracted UC group single-cell sequencing data and determined the proportions of each cell in several division cycles. As shown in Fig. (**S2F**), fibroblasts were mainly active in the M phase and exhibited increased cell division, which could explain the increased proportion of fibroblasts and the observed fiber infiltration score in the UC group.

Finally, using the FindAllMarkers function in the R package “Seurat” to detect fibroblast-associated signature genes with a threshold > 0.25 and padj <0.01, we acquired 700 DEGs related to fibroblasts (Table **S2**).

### 2.3 Identification of Key Fibrosis Genes

We identified the overlapping genes identified from the differential analysis of ScRNA and Bulk-RNA to identify UC-associated fibrosis DEGs. The expression information of DEGs was extracted from the merged UC dataset. We used three types of machine learning methods, SVM-RFE, RF-RFE, and LASSO, to identify key genes using the R package “caret” [13] and select the intersection of the results.

### 2.4 Construction of UC Diagnostic Model

The datasets GSE179285, GSE36807, GSE38713, GSE53306, GSE65114, GSE75214, and GSE87466 were used as the training set. GSE107499 served as the external validation set. The training data was used to build five predictive models using the R package: MLP, RF, SVM, Xgboost, and logistic regression. The optimal model was determined from the external validation set based on the ROC curves and Matthew’s correlation coefficients (MCC). We analyzed the different variables in the best model using the partial dependence profile and SHAP values using R packages “DALEX,” “fastshap,” and “shapviz” [14].

### 2.5 Fibroblast Infiltration and Key Gene Expression in Pediatric UC Samples

The GSE107499 dataset comprised pediatric UC and normal samples. We identified the expression of five hub genes in adults and children in this dataset. In addition, we performed MCP-counter and ESTIMATE scores on this dataset to compare the differences between the four groups. Normality tests were performed on the expression and fiber scores before comparing the groups. Data that met the normal distribution were tested using the ANOVA test; otherwise, the Kruskal-Wallis test was used. A *P*-value corrected by a false discovery rate < 0.01 was considered statistically different.

### 2.6 Prediction of Drug Responsiveness

To assess the value of key genes in clinical applications, we downloaded the infliximab- (GSE12251, GSE14580) [15] and golimumab-treated (GSE92415) [16] UC cohorts from GEO, compared the differences in the expression of key genes between the effective and ineffective groups in the two cohorts, and plotted ROC prediction curves.

### 2.7 Identifying Fibroblast Subtypes

The R package “ConsensusClusterPlus” was used to conduct consensus clustering of UC samples based on hub genes and determine the optimal number of classifications. LASSO regression was used to construct a classification model and calculate the ROC. The R package was used to analyze samples for immune infiltration and compare immune cell differences between subtypes. Next, we compared the differences in the expression of hub genes and inflammatory factors between subtypes. Finally, we conducted single sample gene set enrichment analysis (ssGSEA) using UC samples based on hallmark gene sets and compared the signaling pathways that differed significantly between subtypes. In addition, we divided patients in the infliximab and golimumab treatment cohorts into C1 and C2 subtypes based on the optimal cut-off value and compared the number of responders in the two groups.

### 2.8 Molecular Docking

The ANXA1 protein structure was downloaded from the PDB database. The structures of azathioprine, mesalazine, and tetrandrine were downloaded from Pubchem [17, 18]. PyMOL 2.3.0 software was used to process proteins and small molecules. Molecular docking analysis was conducted using CB-dock2 (https://cadd.labshare.cn/cb-dock2/index.php) [19].

## 3 RESULTS

### 3.1 The Optimal Predictive Model

**Fig. (1):**
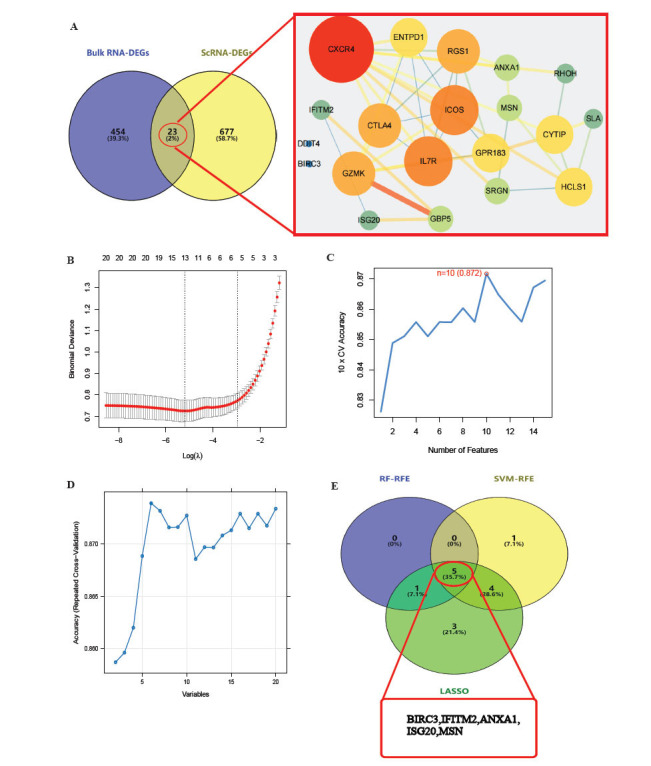
Identification of hub fibrosis-related genes: (**A**) Common fibrosis-related genes: (**B**-**D**) Discovering hub genes *via* Lasso, SVM-RFE, and RF-RFE: (**E**) Five hub genes.

**Fig. (2):**
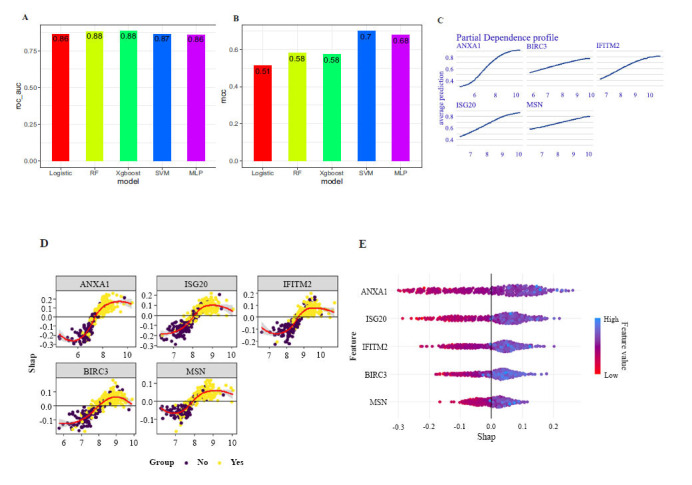
Optimal model selection and interpretability: (**A**, **B**) ROC-AUC and MCC of the models in the external validation set: (**C**) Partial dependence plot of each variable in the optimal model: (**D**, **E**) Degree of contribution of independent variables in the optimal model *via* Shapley value.

Fig. (**1A**) presents the discovery of 23 fibrosis genes linked with UC. We applied three machine learning methods, LASSO, SVM-RFE, and RF-RFE, resulting in the identification of 13, 10, and 6 feature genes, respectively (Figs. **1B**-**1D**). Subsequently, we intersected the results from these three methods to obtain five key genes as independent variables for building models, namely, BIRC3, IFITM2, ANXA1, ISG20, and MSN (Fig. **1E**). After hyperparameter optimization (Figs. **S3A**-**S3D**), all five models yielded an ROC of approximately 0.87 in the external validation set. However, the MCC of SVM was significantly higher than that of the other models, reaching 0.7, indicating the best performance (Figs. **2A** and **2B**). Furthermore, we assessed the degree to which each variable contributes to the SVM model. Fig. (**2C**) shows a nearly linear correlation between the variables and the likelihood of disease, where alterations in ANXA1 expression have a major impact on increasing the probability of disease. As shown in Figs. (**2D** and **2E**), the SHAP values of variables in the UC group were predominantly positive, and the ANXA1 had the most significant impact on the model.

### 3.2 Fibroblast Infiltration and Expression of Key Genes in Pediatric UC Samples

As shown in Figs. (**3A** and **3B**), fibroblast infiltration scores were not significantly different between the adult and pediatric UC groups, nor between the adult and pediatric normal groups (*P*>0.05). However, fibrosis scores in the pediatric UC group were higher than those in the normal pediatric group (*P*<0.01). Fig. (**3C**) depicts that the expression of none of the five hub genes was significantly different between the pediatric UC and adult UC groups. Except for BIRC3, the expression of the four hub genes was not significantly different between the adult and pediatric normal groups.

### 3.3 Predicting Efficacy in UC Patients Treated With TNFα Inhibitors

As shown in Figs. (**4A**-**4B**), in the golimumab and infliximab cohorts, the expression of the five hub genes was significantly higher in the ineffective group than in the effective group (*P*<0.05). The ROCs for predicting treatment efficacy for each of the five biomarkers were ≥0.6. he number of individuals exhibiting high expression levels was significantly greater (*P*<0.05) in the null group when divided into two subgroups, high and low, based on the median value of ANAX1.

### 3.4 Fibroblast Subtype Characterization

As shown in Figs. (**S4A**-**S4C**), unsupervised clustering identified two subtypes of UC, and PCA confirmed this result. The LASSO binary classification model was constructed for both subtypes, and all five hub genes were included as variables in the model. Based on the obtained correlation coefficients, we computed the fiber cell score as follows: Score=0.406*exp(IFITM2)-1.352*exp(BIRC3)+0.309*exp(ANXA1)-0.131*(ISG20)+0.712*(MSN). The ROC of the binary classification model reached 0.994, and the optimal cutoff value was -0.503, indicating that values greater than the cutoff are attributed to the C2 subtype; otherwise, they belong to the C1 (Figs. **S4D**-**S4F**).

**Fig. (5):**
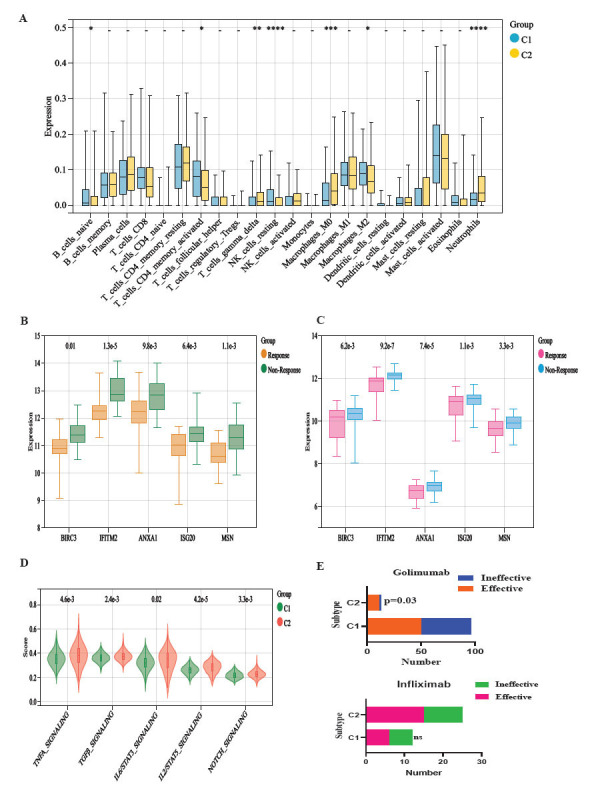
Immunological characterization of fiber gene subtypes: (**A**) Differential analysis of immune cells: (**B**) Differential analysis of fibrosis-related cytokines: (**C**) Differential analysis of 5 hub fibrosis-related genes: (**D**) ssGSEA: (**E**) TNFα inhibitor effectiveness in different subtypes.

We also compared the differences between the two subtypes of immune cells. The proportion of B-cell memory, T-cells CD4 memory activated, macrophages M2, and NK resting cells was higher in the C1 subtype; the proportion of T-cells gamma delta and neutrophils was higher in the C2 subtype (Fig. **5A**).

The expression of the five hub genes, as well as the fibrosis-associated inflammatory factors, TNF, IL33, IL11, and ICAM1, were significantly elevated in the C2 subtype (Figs. **5B** and **5C**). The ssGSEA results showed that inflammatory pathways, such as TNF, IL6-STAT3, and IL2-STAT5, as well as fibrotic signaling pathways, such as TGFβ and Norch, were significantly increased in the C2 phenotype (Fig. **5D**). Among the patients who received golimumab treatment, there were 13 people identified as belonging to the C2 subtype. Among these, 11 patients exhibited a favorable response to the medication. However, 2 patients did not demonstrate any improvement. There were 96 individuals categorized as belonging to the C1 subtype. Out of these, 50 had a successful reaction, and 46 had an ineffective response. In contrast, patients with the C2 subtype demonstrated a greater positive response to golimumab. Out of the patients who received infliximab treatment, 12 individuals were categorized as subtype C1. Among them, 6 patients exhibited a positive response to the treatment, while the remaining 6 patients did not demonstrate any improvement. Among the entire sample, 25 were categorized as subtype C2, with 15 demonstrating advantageous effects and 10 showing ineffectiveness. Although the difference in the ratio of effective and ineffective numbers across different types of infliximab was not statistically significant, the C2 subtype group had a higher number of persons who responded positively (Fig. **5E**).

### 3.5 Molecular Docking of ANXA1 to Small Molecule Drugs

We used CB-dock2 for conducting molecular docking to the target proteins. Binding energy < -5 kcal/mol indicates good binding, while < -7 kcal/mol indicates strong binding. We found that ANXA1 binds to azathioprine, mesalazine, and tetrandrine with binding energies of -5.5 kcal/mol, -6.5 kcal/mol, and -7.2 kcal/mol, respectively. The docking results are visualized in Fig. (**S5**).

## 4 DISCUSSION

The association between intestinal fibrosis and Crohn’s disease has been widely accepted. Unfortunately, UC-associated intestinal fibrosis remains poorly explored. Extracellular matrix deposits transgress the full thickness of the colonic wall in patients with UC, which may lead to anal dysfunction and fecal incontinence [20]. In a comparative study involving subjects with UC, colorectal cancer, and normal regional tissues, submucosal fibrosis was identified in all specimens collected from UC patients. Furthermore, submucosal fibrosis and thickening of the mucosal muscular layer were observed in conjunction with chronic inflammation but not with active inflammation [21]. Thus, both fibrosis and thickening of the mucosal muscular layer were suggested as common complications of progressive UC. Another study revealed persisting abnormalities, including ECM remodeling, increased production of pro-fibrotic cytokines, and activated TGF-β signaling pathway, even in endoscopically normal UC mucosa [22]. These findings imply that while inflammation is a prerequisite in fibrogenesis in UC, suppressing inflammation and subsequent healing does not necessarily prevent fibrosis development. In this study, we integrated eight bulk-RNA UC datasets using four immune infiltration analyses. The degree of fibroblast infiltration and the stromal score were significantly higher in the UC group than in the normal group. Moreover, UC fibroblast infiltration and stromal scores were significantly higher in the pediatric UC group than in the normal pediatric group. In addition, we analyzed the ScRNA dataset and found that the proportion of fibroblasts was significantly higher in the UC group than in the normal group. Furthermore, in the UC group, the fibroblasts were active and mainly in the mitotic phase.

Five hub genes, namely ANXA1, BIRC3, IFITM2, ISG20, and MSN, were identified in this study. ANXA1, contributing most to the diagnostic model of UC, is a calcium-dependent phospholipoprotein, localized on the cell membrane; it is regulated by glucocorticoids and can inhibit cytoplasmic phospholipase A2 and block the release of arachidonic acid [23, 24]. ANXA1 has an anti-inflammatory effect, inhibits neutrophil recruitment, and promotes phagocytosis by macrophages [25]. Studies have reported increased ANXA1 expression in inflammatory tissues of UC patients with milder clinical disease activity [26]. ANXA1 knockout mice (AnxA-/-) exhibited higher susceptibility to DSS-induced colitis [27]. Currently, ANXA1-based nano-targeted delivery drugs have been developed for the treatment of UC [28]. However, intestinal mucosal ANXA1 levels were higher in UC patients in this study. A study reported that the addition of human recombinant ANXA1 (hrANXA1) to T cells after stimulation with CD3 and CD28 enhanced T cell proliferation and activation [29]. Colitis was induced by transplantation of naïve CD4^+^ T cells in immunodeficient mice. After 5-10 weeks, the expressions of type I and type III collagen and TGF-β in the mouse colon were significantly increased, and the intestinal tissue became fibrotic [30]. Moreover, in colorectal cancer samples, high ANXA1 expression promoting macrophage polarization towards an M2-like phenotype is associated with immunosuppression and strongly correlated with poor prognosis [28]. In summary, ANXA1 likely serves a dual role in UC. On the one hand, it exerts a protective function against colitis and inhibits the spread of inflammation. On the other hand, it acts as an intestinal fibrosis risk factor, limiting its application in anti-UC. It is essential to thoroughly assess the benefits and drawbacks of ANXA1 for UC to ensure treatment effectiveness and safety. BIRC3 is a multifunctional protein that regulates not only cysteinyl asparagine and apoptosis but also inflammatory signaling, immunity, pro-mitotic kinase signaling, and cell proliferation [31].

BIRC3 promotes anti-apoptotic effects by binding to TNFR2 and further activating NF-κB and MAPK pathways [32]. Animal models of periodontitis and rheumatoid arthritis showed upregulated BIRC3 expression, which effectively attenuated inflammatory necrosis [33, 34]. Several esophageal cancer cell lines also showed upregulated BIRC3 and activation of the Wnt and fibroblast growth factor signaling pathways [35]. Therefore, BIRC3 expression may be upregulated in UC patients and promote intestinal fibrosis. Unfortunately, there is currently no direct evidence in this regard, and further studies are warranted. IFITM2 belongs to a small family of interferon-stimulated proteins with molecular weights of 10-20 kDa; it is found in primates [36, 37]. IFITM2 is involved in the osteogenic differentiation of C3H10T1/2 cells. It is upregulated during osteogenic differentiation and Ifitm2 knockdown results in a decrease in osteogenic markers. Transcriptome analysis revealed the involvement of Wnt/β-catenin signaling in IFITM2 overexpression, suggesting a potential interaction of Ifitm2 with the classical Wnt signaling pathway that contributes to osteogenic differentiation [38]. Single-cell sequencing analysis of skin tissue from patients with juvenile dermatomyositis, which is an autoimmune disease, revealed a significant increase in the proportion of fibroblasts compared to normal tissue and increased IFITM2 expression in monocytes and myofibroblasts [39]. This suggests interactions between inflammatory cells and fibroblasts. In the present study, in line with previous studies, we found significantly increased fibroblasts and IFITM2 levels in UC tissues in our single-cell sequencing results, suggesting that IFITM2 is involved in UC-associated intestinal fibrosis. However, the precise mechanism behind this potential phenomenon must be further investigated.

ISG20 belongs to the family of interferon-stimulated genes and activates T cells to exert antiviral effects. In recent years, ISG20 has been involved in the progression of various tumors, such as cervical cancer, kidney cancer, and glioma. In renal clear cell carcinoma, ISG20 positively regulates the expression of CCND1, which promotes cell proliferation and upregulates the expression of MMP9, which is involved in the breakdown of the extracellular matrix and promotes metastasis [40-42]. MSN is a member of the ezrin-radixin-moesin (ERM) family and plays an important role in mediating the binding of F-actin to the plasma membrane and is, therefore, involved in several cellular functions, such as cell migration, growth, adhesion, cell polarization, motility, and fibrosis [43]. In a model of liver injury-associated fibrosis, MSN promoted collagen and actin expression through the MRTF-A signaling pathway. MSN knockdown also attenuated liver fibrosis [44]. Hence, ISG20 and MSN were found to be potentially involved in UC-associated intestinal fibrosis.

We also identified two fibroblast subtypes by unsupervised clustering of the five hub genes. The C2 subtype showed higher expression of the five fibroblast-related genes than the C1 subtype. The C2 subtype showed higher expression of five fibroblast genes than the C1 subtype. Furthermore, the levels of inflammatory factors, such as TNFα, IL33, IL11, and ICAM1, were higher in the C2 subtype than in the C1 subtype. TNFα is a recognized major cytokine involved in the development of IBD and is involved in mucosal inflammation. The involvement of TNFα in intestinal fibrosis has not been fully elucidated. Theiss *et al.* found that TNFα binds to IGF-I, a growth factor that is a key pro-fibrotic during intestinal inflammation *in vivo* and promotes collagen synthesis. TNFα activates the TNFR2 receptor to stimulate the proliferation of myofibroblasts, mediated by the ERK1/2 signaling pathway [45]. IL33 belongs to the IL1 family of cytokines. It not only induces Th2 cells but also activates TH1 cells and Treg cells [46]. UC patients have been shown to have increased local intestinal mucosal IL-33 levels, which decreased after treatment with infliximab [47]. A mouse model of DSS-induced colitis demonstrated that intestinal epithelial cells and fibroblasts locally produce IL-33, which promotes mucosal healing *via* the IL-33-ST2 axis [48]. IL33 has a dual role in UC progression and works by activating the NF-κB-p65 pathway to act as a pro-inflammatory factor, as well as activating fibroblasts for tissue repair.

## CONCLUSION

In summary, our study revealed increased fibroblast infiltration and stromal scores in UC, particularly in pediatric cases, thus emphasizing the role of fibrosis in disease progression. ANXA1, BIRC3, IFITM2, ISG20, and MSN likely contribute to UC pathogenesis, including inflammation and fibrosis, and must be further investigated. Our findings provide insights into potential therapeutic targets for mitigating fibrosis in UC.

## Figures and Tables

**Fig. (3):**
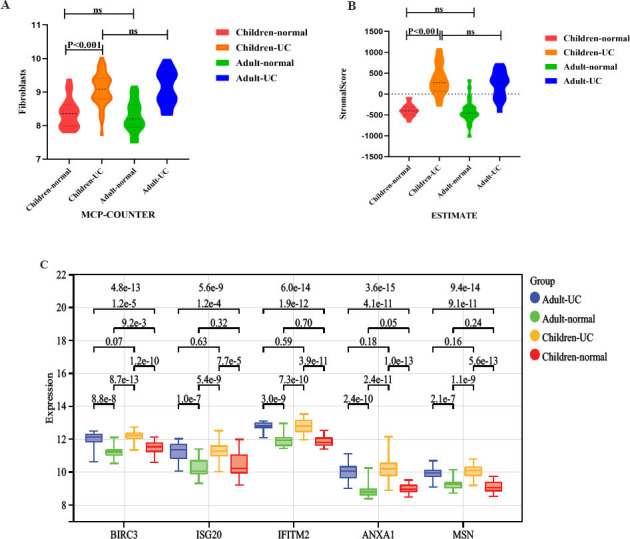
Fibroblast infiltration and hub genes in children with UC differ from those in normal children. (**A**-**B**) Fibrocystic infiltration score: (**C**) Differences in 5 hub genes between children and adult samples.

**Fig. (4):**
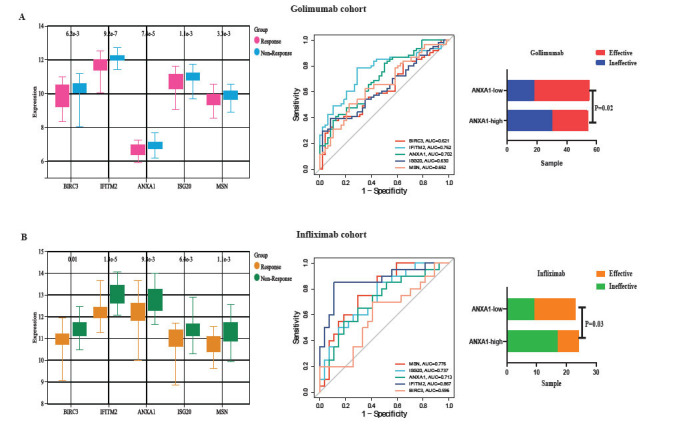
Five hub genes predict the effectiveness of TNF inhibitors in the treatment of UC. (**A**) Golimumab. (**B**) Infliximab.

## Data Availability

This study analyzed the published data sets. These data can be found in the Gene Expression Omnibus (http://www.ncbi.nlm.nih.gov/geo).

## LIST OF ABBREVIATIONS

UC: Ulcerative Colitis
RF: Random Forest
SVM: Support Vector Machine
MLP: Multilayer Perceptron
MCC: Matthew’s Correlation Coefficient
ssGSEA: Single Sample Gene Set Enrichment Analysis
ERM: Ezrin-Radixin-Moesin
