# Supplementary material for: Revealing Fibrosis Genes as Biomarkers of Ulcerative Colitis: A Bioinformatics Study Based on ScRNA and Bulk RNA Datasets
Source: Endocr Metab Immune Disord Drug Targets. 2024 Oct 18;25(9):710–20. doi: 10.2174/0118715303332155240912050838 (PMC12376109; doi:10.2174/0118715303332155240912050838)
Supplement: Supplementary file 1 [file EMIDDT-25-9-710_SD1.pdf]

Supplementary Material

Revealing Fibrosis Genes as Biomarkers of Ulcerative Colitis: A Bioinformatics Study Based on ScRNA and Bulk RNA Datasets

Yandong Wang[1], Li Liu[2] and Weihao Wang[3,*]

[1]College of Nursing and Rehabilitation, North China University of Science and Technology, Tangshan, 063000, China; [2]North China University of Science and Technology Affiliated Hospital, Intensive Care Medicine Department, Tang-shan, 063000, China; [3]School of Chemical and Biological Engineering, Yichun University, Yichun, 336000, Jiangxi,China

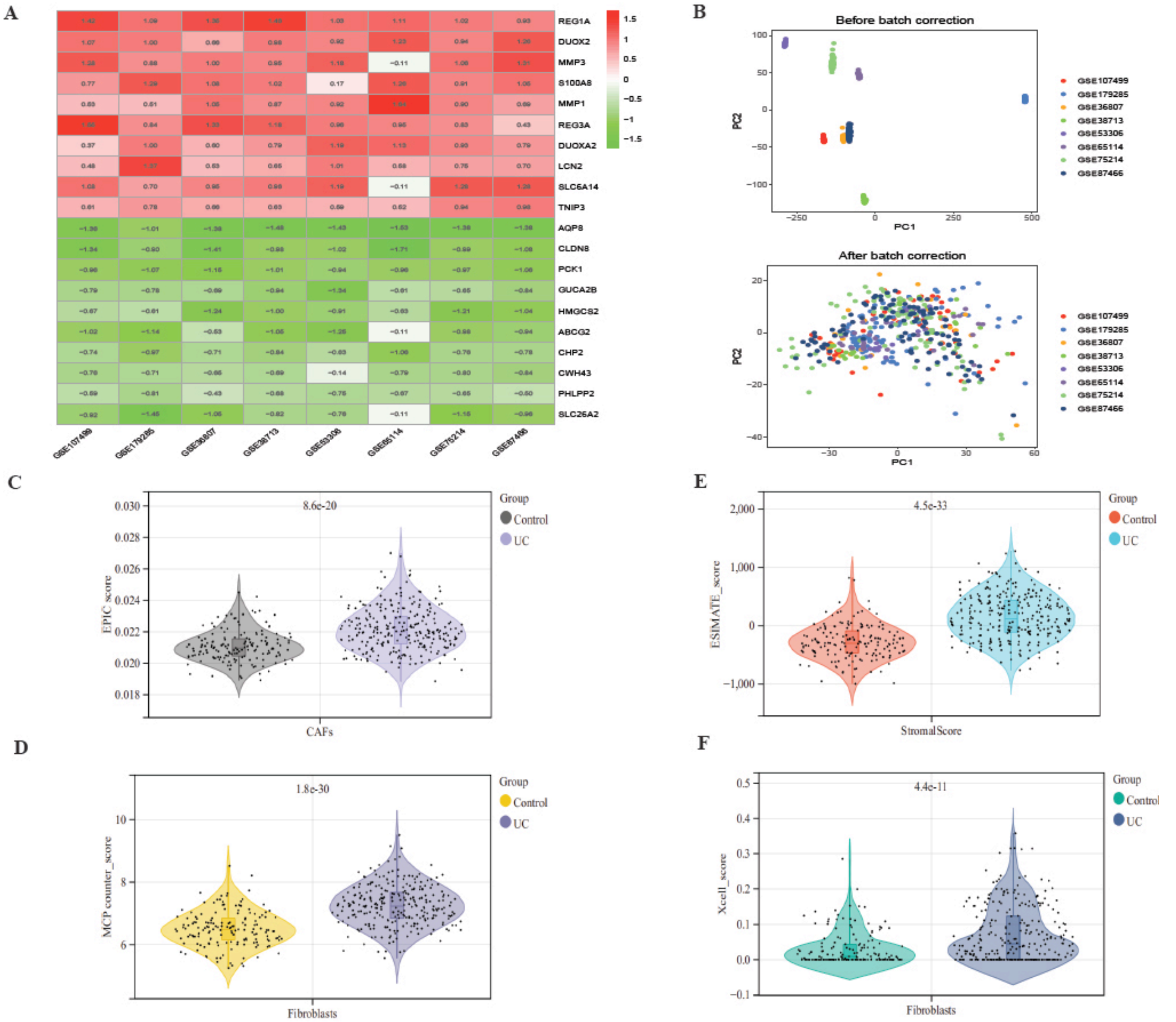

Fig. S1. Data processing and fibroblast infiltration for bulk-RNA datasets. (**A**) Differentially expressed gene. (**B**) Removing batch effect. (**D**) Xcell. (**E**) EPIC. (**F**) MCP_counter. (**G**) ESTIMATE.

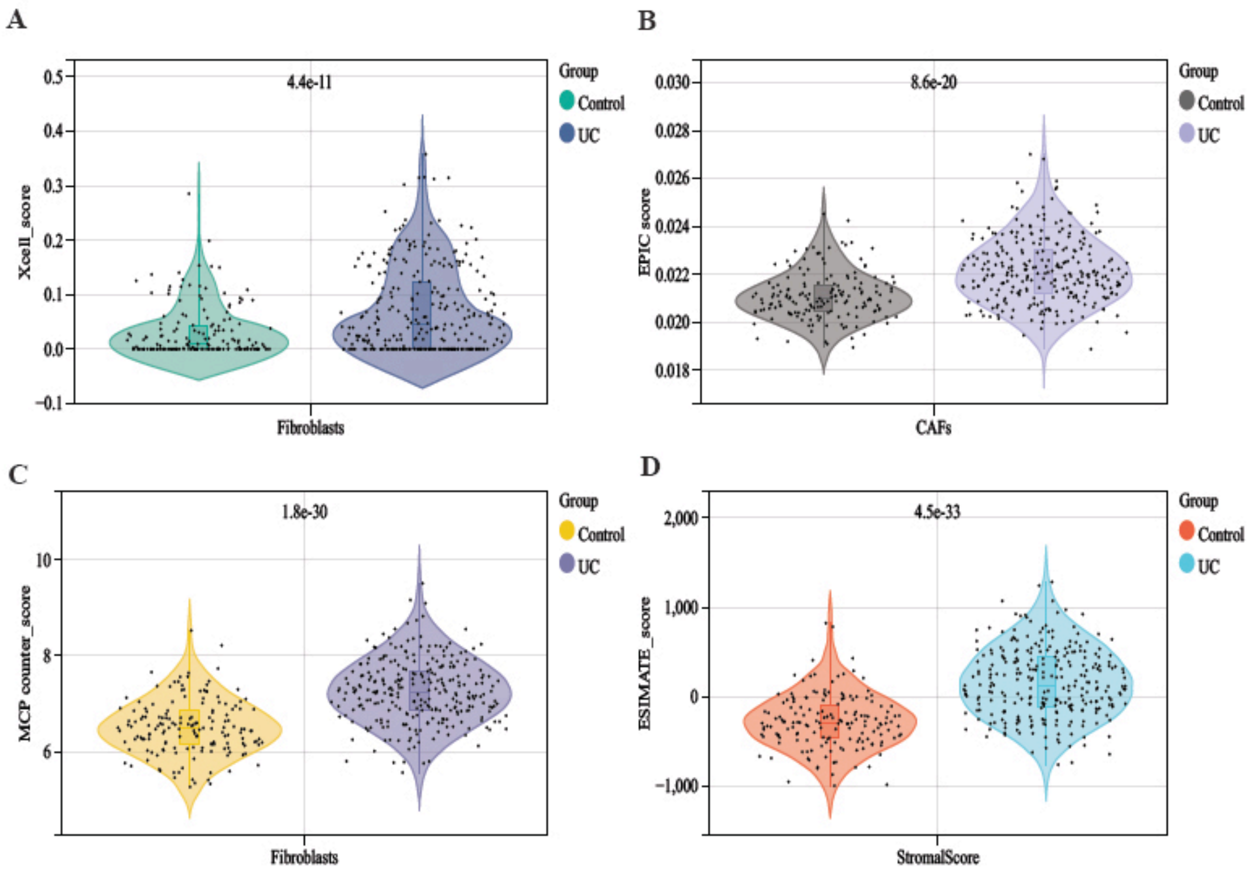

Fig. S2. Analysis of single-cell sequencing data. (**A**) Removing batch effect. (**B**) Elbow plot. (**C-D**) Dimensionality reduction, clustering, and cellular annotation. (**E**) Differences in the proportion of fibroblasts. (**F**) Mitotic cycle.

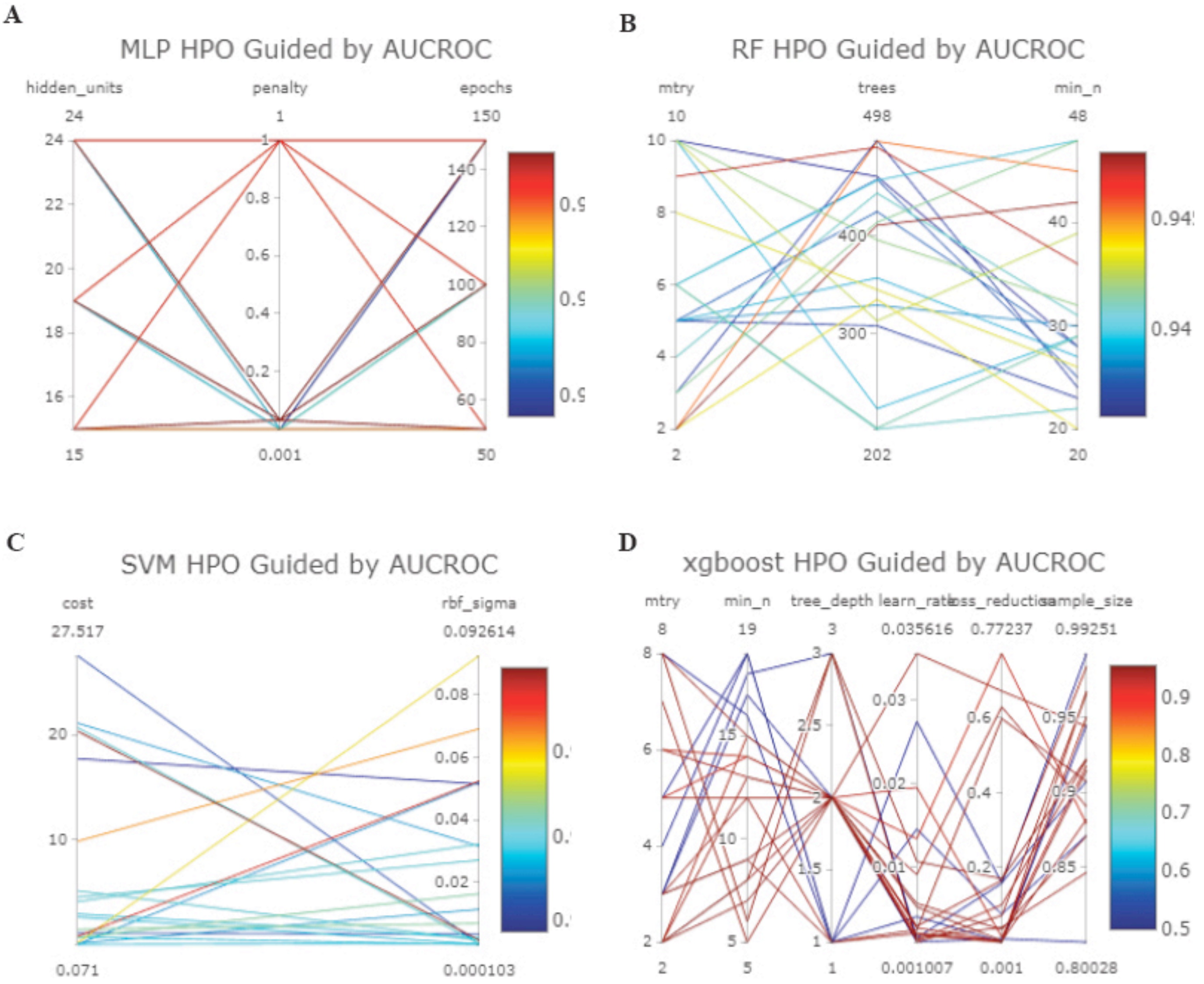

Fig. S3. Visualization of hyperparameter optimization results for machine learning models. (**A**) MLP. (**B**) RF. (**C**) SVM. (**D**) Xgboost.

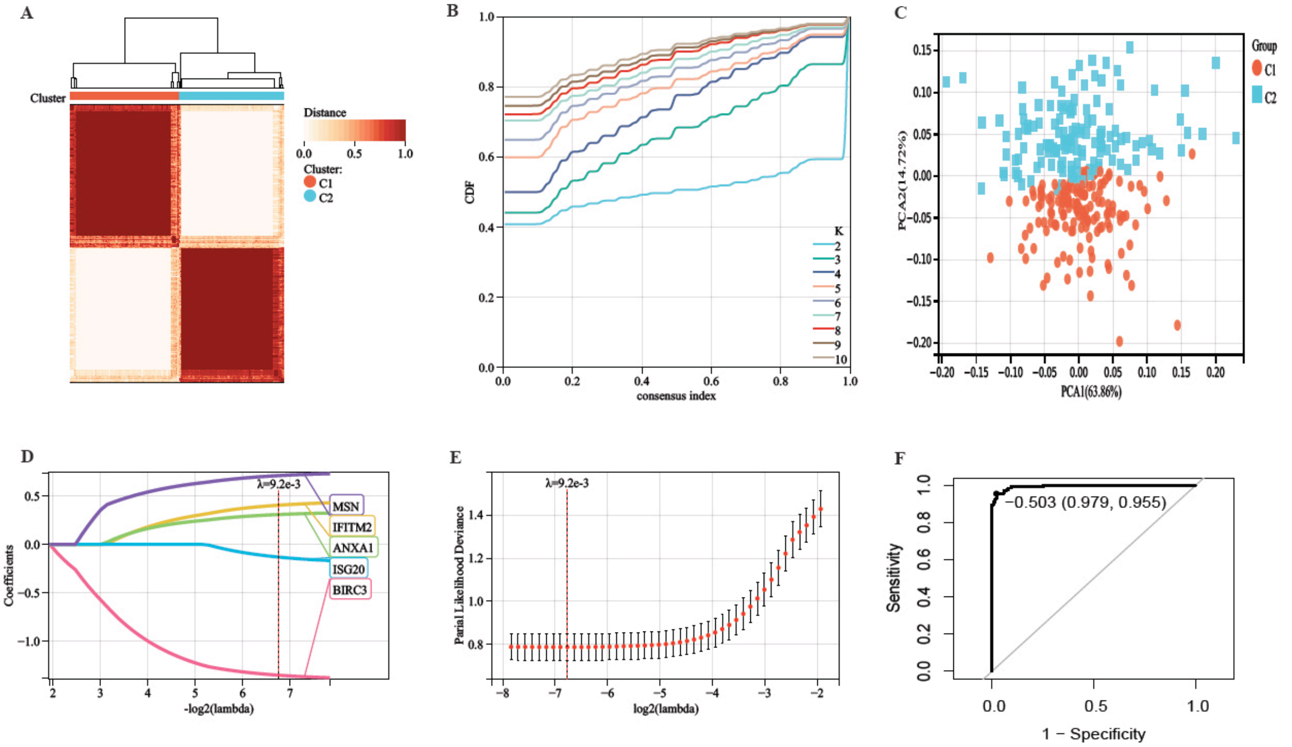

Fig. S4. Fibrosis genes subtype. (**A-B**) Unsupervised clustering. (**C**) PCA. (**E-F**) Classification model based on 5 hub gene signature.

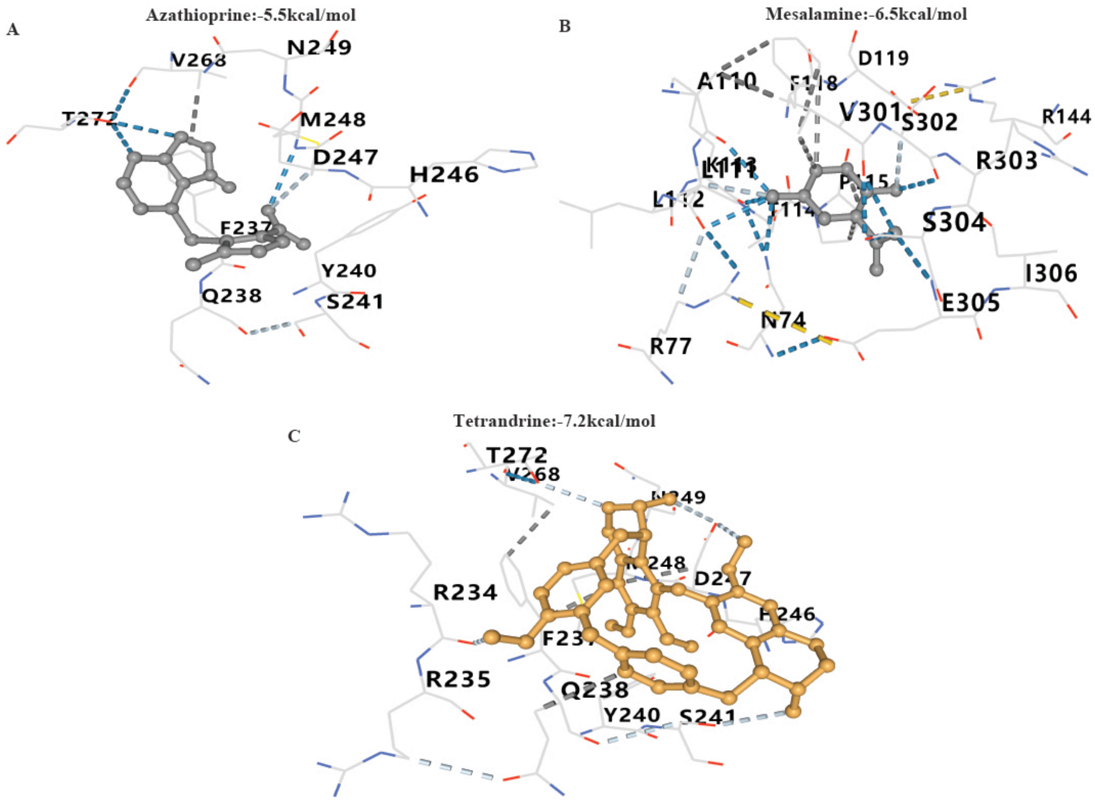

Fig. S5. Molecular docking.

Table S1: The results of RRA in bulk-RNA datasets.

Name	*p*-value	Fdr	LogFC
AQP8	1.52E-27	4.89E-23	-4.45964
REG1A	1.82E-26	5.85E-22	4.772252
DUOX2	2.69E-25	4.31E-21	3.949788
CLDN8	7.83E-25	1.26E-20	-3.60919
PCK1	3.56E-23	3.8E-19	-3.13494
MMP3	1.12E-21	1.13E-17	3.992365
S100A8	1.63E-21	1.13E-17	3.808227
MMP1	1.76E-21	1.13E-17	3.564873
REG3A	6.21E-21	3.32E-17	4.129818
DUOXA2	1.11E-20	4.78E-17	3.324619
LCN2	1.19E-20	4.78E-17	2.95499
GUCA2B	7.91E-21	6.35E-17	-2.4781
HMGCS2	1.32E-20	8.46E-17	-2.99295
SLC6A14	2.46E-20	8.78E-17	3.964004
TNIP3	7.11E-20	2.28E-16	3.018234
MMP10	1.32E-19	3.84E-16	2.891052
DMBT1	1.64E-19	4.38E-16	2.749416
REG1B	2.05E-19	5.06E-16	4.253663
CXCL11	4.52E-19	1.04E-15	2.489577
ABCG2	2.05E-19	1.1E-15	-2.72614
PI3	1.07E-18	2.29E-15	2.618959
CHP2	6.81E-19	3.12E-15	-2.35091
CWH43	1.07E-18	4.29E-15	-1.97192
REG4	2.44E-18	4.71E-15	2.340219
MMP12	2.49E-18	4.71E-15	2.389289
CHI3L1	3.05E-18	5.44E-15	3.543416
PHLPP2	2.25E-18	7.49E-15	-1.71179
SLC26A2	2.33E-18	7.49E-15	-2.77903
S100A9	1.06E-17	1.8E-14	2.276866
VNN1	1.36E-17	2.18E-14	2.517804
MMP7	2.01E-17	3.08E-14	2.45596
CAPN13	1.26E-17	3.51E-14	-1.74332
UGT2A3	1.31E-17	3.51E-14	-2.50952
IDO1	3.5E-17	5.1E-14	2.641118
CXCL1	3.91E-17	5.46E-14	3.054647
TRPM6	3.5E-17	8.63E-14	-2.22454
TSPAN7	4.19E-17	9.61E-14	-1.35459
CXCL9	1.7E-16	2.27E-13	2.454131
SELP	2.74E-16	3.51E-13	1.847876
AQP9	3.01E-16	3.62E-13	2.283983
IL1B	3.12E-16	3.62E-13	2.373372
CXCL8	3.16E-16	3.62E-13	2.384963

CXCL13	3.78E-16	4.18E-13	2.117744
RUNDC3B	2.39E-16	5.12E-13	-1.55889
MMP9	5.8E-16	6.2E-13	1.94798
TCN1	6.16E-16	6.38E-13	2.639843
SELL	6.52E-16	6.54E-13	1.858441
IL33	1.02E-15	9.96E-13	1.419293
SLC17A4	5.46E-16	1.1E-12	-1.71009
CA1	6.06E-16	1.14E-12	-2.0849
FMO5	6.38E-16	1.14E-12	-1.37613
DHRS11	6.91E-16	1.17E-12	-1.69273
FCRL5	1.39E-15	1.32E-12	1.430748
SOCS3	1.6E-15	1.47E-12	1.667703
PBLD	1.02E-15	1.64E-12	-1.43712
S100A12	1.92E-15	1.71E-12	2.172217
CDHR1	1.2E-15	1.84E-12	-1.43587
PIM2	2.5E-15	2.17E-12	1.618076
DPP10	1.49E-15	2.17E-12	-1.69453
MT1M	1.71E-15	2.39E-12	-2.14578
IL1RN	2.9E-15	2.44E-12	2.187316
NCF2	2.96E-15	2.44E-12	1.556742
GBP5	3.11E-15	2.49E-12	1.565287
CXCL10	3.29E-15	2.58E-12	2.270351
SERPINB5	3.73E-15	2.85E-12	2.322187
ENTPD5	2.14E-15	2.87E-12	-1.48566
LAX1	4.75E-15	3.55E-12	1.563122
SGK2	3.73E-15	4.79E-12	-1.5571
HHLA2	6.25E-15	7.72E-12	-1.11057
SLC16A9	7.01E-15	8.33E-12	-1.78931
NOS2	1.2E-14	8.76E-12	2.110646
S100P	1.26E-14	8.99E-12	1.6748
TMEM37	8.16E-15	9.35E-12	-1.3609
CCL11	1.45E-14	1.01E-11	1.832529
RHOU	9.63E-15	1.07E-11	-1.35729
CKB	1.07E-14	1.11E-11	-1.49594
MEP1A	1.07E-14	1.11E-11	-1.46009
HEPACAM2	1.3E-14	1.3E-11	-1.51758
CTSK	2.02E-14	1.38E-11	1.496605
C2orf88	1.55E-14	1.51E-11	-1.2526
FPR1	2.29E-14	1.53E-11	1.689404
VIPR1	1.81E-14	1.65E-11	-1.39207
ANO5	1.81E-14	1.65E-11	-1.14325
GBA3	1.85E-14	1.65E-11	-1.91421
DEFA5	2.62E-14	1.71E-11	3.249824
MZB1	2.67E-14	1.71E-11	1.415158
BEST2	2.02E-14	1.75E-11	-1.76091
GREM1	2.86E-14	1.8E-11	1.90762

DEFA6	3.25E-14	2.01E-11	2.870542
ANKRD22	3.36E-14	2.03E-11	1.299857
TIMP1	3.85E-14	2.29E-11	1.921444
PDE6A	2.76E-14	2.32E-11	-1.69618
EDN3	2.82E-14	2.32E-11	-1.23172
PROK2	4.23E-14	2.46E-11	1.885995
ASPHD2	4.29E-14	2.46E-11	1.156556
ABCB1	3.35E-14	2.69E-11	-1.85569
PPARG	3.8E-14	2.97E-11	-1.24885
CATSPERB	5.37E-14	3.03E-11	1.349034
TFF1	5.78E-14	3.2E-11	1.351937
DERL3	6.04E-14	3.23E-11	1.446055
CFB	6.04E-14	3.23E-11	1.738674
CLYBL	4.43E-14	3.38E-11	-1.00714
PAQR5	4.98E-14	3.71E-11	-1.23715
SELENBP1	5.3E-14	3.86E-11	-1.42153
PECAM1	8.26E-14	4.35E-11	1.511653
ACSF2	1.02E-13	7.25E-11	-1.60084
SAMD9L	1.42E-13	7.33E-11	1.361536
BMP3	1.13E-13	7.9E-11	-1.58134
IGFBP5	1.57E-13	8.01E-11	1.882404
CCL19	1.63E-13	8.19E-11	1.439089
KYNU	1.71E-13	8.43E-11	2.206861
MT1F	1.31E-13	8.92E-11	-1.26528
SERPINA3	1.92E-13	9.34E-11	2.186707
GEM	2.03E-13	9.71E-11	1.287675
KDELR3	2.1E-13	9.93E-11	1.38469
OLFM4	2.27E-13	1.05E-10	1.982624
C4BPB	2.38E-13	1.09E-10	1.93973
MYEOV	2.44E-13	1.1E-10	1.38188
SLC30A10	1.9E-13	1.27E-10	-1.76966
CNTN3	2.05E-13	1.34E-10	-1.79122
TUBAL3	2.38E-13	1.53E-10	-1.43629
DAPP1	3.6E-13	1.59E-10	1.480134
CDH3	3.61E-13	1.59E-10	1.856746
CNGA1	2.53E-13	1.59E-10	-1.28546
LDHD	2.59E-13	1.6E-10	-1.10344
CFI	3.69E-13	1.6E-10	1.791632
ADH1C	2.69E-13	1.63E-10	-1.86858
GUCA2A	2.89E-13	1.72E-10	-1.91914
CCL18	4.29E-13	1.84E-10	1.643782
PPARGC1A	3.28E-13	1.91E-10	-1.41961
LRRC19	3.36E-13	1.92E-10	-1.35809
HSD17B2	3.45E-13	1.94E-10	-1.41342
C4BPA	4.96E-13	2.1E-10	2.027961
METTL7B	3.83E-13	2.12E-10	-1.15057

PRR15	4.05E-13	2.2E-10	-1.09846
CCR7	5.67E-13	2.36E-10	1.261076
PTGDR	4.44E-13	2.38E-10	-1.38061
NAALADL1	4.65E-13	2.45E-10	-1.24927
CA7	4.97E-13	2.54E-10	-1.17448
PTPRR	4.98E-13	2.54E-10	-1.271
PNLIPRP2	5.23E-13	2.59E-10	-1.63032
IGSF9	5.33E-13	2.59E-10	-1.19258
NAAA	5.55E-13	2.62E-10	-1.14341
EXPH5	5.69E-13	2.65E-10	-1.51523
ANK3	5.82E-13	2.67E-10	-1.29518
CD38	6.49E-13	2.67E-10	1.551015
PCSK1	6.64E-13	2.7E-10	1.60621
PADI2	6.72E-13	3.04E-10	-1.72202
PDZD3	7.35E-13	3.25E-10	-1.21133
ARHGAP44	7.39E-13	3.25E-10	-1.10646
PLA1A	9.06E-13	3.59E-10	1.466829
MNDA	9.06E-13	3.59E-10	1.526292
STC1	9.3E-13	3.64E-10	1.461582
CXCL6	1.04E-12	4E-10	2.243008
ISX	9.9E-13	4.23E-10	-1.27981
FGFR2	1E-12	4.24E-10	-1.23175
APOL1	1.21E-12	4.62E-10	1.379241
AHCYL2	1.12E-12	4.68E-10	-1.02773
SCUBE2	1.23E-12	4.95E-10	-1.20306
EDN1	1.23E-12	4.95E-10	-1.09754
TDO2	1.36E-12	5.08E-10	1.696479
LAMP3	1.36E-12	5.08E-10	1.469913
BEST4	1.31E-12	5.13E-10	-1.32925
CDKN2B	1.31E-12	5.13E-10	-1.32157
MAOA	1.59E-12	6.15E-10	-1.1771
SERPINB7	1.68E-12	6.21E-10	1.733058
CXCL3	1.71E-12	6.25E-10	1.946256
ETFDH	1.65E-12	6.3E-10	-1.02706
MEP1B	1.71E-12	6.45E-10	-1.70547
ABAT	1.73E-12	6.45E-10	-1.03784
CD27	1.81E-12	6.54E-10	1.203066
CXCL2	1.96E-12	6.95E-10	1.940096
GZMB	1.97E-12	6.95E-10	1.617963
MT1G	1.92E-12	7.08E-10	-1.23052
GXYLT2	1.97E-12	7.1E-10	-1.33146
SLC22A5	1.97E-12	7.1E-10	-1.44976
SCIN	2.07E-12	7.37E-10	-1.26453
CCL20	2.35E-12	8.19E-10	1.380818
CXCL5	2.39E-12	8.25E-10	2.162535
PKIB	2.41E-12	8.5E-10	-1.32665

BACE2	2.68E-12	9.13E-10	1.228107
PDPN	2.81E-12	9.48E-10	1.453178
IL13RA2	2.93E-12	9.8E-10	1.792998
DEPDC7	2.96E-12	1.03E-09	-1.13422
LTF	3.17E-12	1.05E-09	1.094853
TRIM22	3.23E-12	1.06E-09	1.254866
TNC	3.64E-12	1.18E-09	1.932142
SPNS2	4.12E-12	1.32E-09	1.247442
CD177	3.88E-12	1.32E-09	-1.58736
CHI3L2	4.6E-12	1.46E-09	1.272154
GPR160	4.68E-12	1.58E-09	-1.02183
FMO4	4.9E-12	1.62E-09	-1.15171
CHRDL2	5.21E-12	1.64E-09	1.300852
PLAU	5.26E-12	1.64E-09	1.785032
SLA	6.13E-12	1.89E-09	1.137267
CNKSR3	5.85E-12	1.91E-09	-1.0721
CHAD	5.9E-12	1.91E-09	-1.26681
VNN2	6.33E-12	1.93E-09	1.357563
PDZK1IP1	6.43E-12	1.95E-09	1.478529
PLA2G12B	6.23E-12	2E-09	-1.44059
PIGZ	6.46E-12	2.05E-09	-1.24417
SRGN	7.06E-12	2.12E-09	1.177702
HPGD	6.84E-12	2.13E-09	-1.04353
TAGAP	7.52E-12	2.23E-09	1.130931
SATB2	7.39E-12	2.28E-09	-1.15654
SLC4A4	7.62E-12	2.33E-09	-1.3295
ZNF704	7.88E-12	2.39E-09	-1.08456
C2	8.26E-12	2.41E-09	1.417053
THY1	8.47E-12	2.44E-09	1.218721
ADM	8.53E-12	2.44E-09	1.017556
EFNA1	8.34E-12	2.48E-09	-1.16974
C7orf31	8.34E-12	2.48E-09	-1.16557
GZMK	8.86E-12	2.49E-09	1.296676
ANPEP	8.6E-12	2.51E-09	-1.49427
GALNT12	8.86E-12	2.56E-09	-1.02547
ANGPTL2	9.58E-12	2.67E-09	1.227971
A1CF	9.4E-12	2.69E-09	-1.0251
OLFML2B	9.8E-12	2.71E-09	1.132308
ACVR1C	1.08E-11	3.07E-09	-1.04044
PRRX1	1.22E-11	3.35E-09	1.60324
AIM2	1.27E-11	3.47E-09	1.21148
SLC3A1	1.33E-11	3.74E-09	-1.34423
NNMT	1.45E-11	3.84E-09	1.36581
SPP1	1.45E-11	3.84E-09	1.692339
IGFBP7	1.58E-11	4.16E-09	1.007085
HTRA1	1.81E-11	4.73E-09	1.223266

SPINK4	1.93E-11	4.98E-09	2.038877
SAMD13	1.9E-11	5.08E-09	-1.08773
TREM1	2.35E-11	6.02E-09	1.188626
CLDN1	2.4E-11	6.1E-09	1.382419
MTMR11	2.74E-11	7.08E-09	-1.00799
C3	2.85E-11	7.19E-09	1.691914
SPAG4	2.92E-11	7.31E-09	1.207387
SLC16A1	3E-11	7.7E-09	-1.27386
LEAP2	3.43E-11	8.53E-09	-1.01688
THBS2	3.56E-11	8.71E-09	1.286897
FKBP11	3.56E-11	8.71E-09	1.201892
SATB2-AS1	3.54E-11	8.74E-09	-1.38392
GPX7	3.68E-11	8.93E-09	1.096138
RHOH	3.7E-11	8.93E-09	1.101102
KLK10	4.13E-11	9.89E-09	1.670458
USP30	4.1E-11	9.98E-09	-1.0039
PTGDS	4.42E-11	1.05E-08	1.310968
BIRC3	4.63E-11	1.09E-08	1.211595
GBP4	5.06E-11	1.18E-08	1.046014
C1orf210	5.09E-11	1.2E-08	-1.01837
C1R	5.43E-11	1.26E-08	1.220837
SELE	5.56E-11	1.28E-08	1.317493
CYTIP	5.71E-11	1.3E-08	1.090645
TRIM29	6.46E-11	1.46E-08	1.394593
MCOLN2	6.54E-11	1.48E-08	-1.08389
SLAMF7	6.83E-11	1.53E-08	1.086374
HAPLN3	7.61E-11	1.69E-08	1.196645
FAP	7.8E-11	1.7E-08	1.325144
HAVCR1	7.97E-11	1.76E-08	-1.17401
TMED6	8.06E-11	1.77E-08	-1.07033
ICAM1	8.88E-11	1.9E-08	1.248816
PLEKHG6	8.98E-11	1.95E-08	-1.0211
MS4A12	9.17E-11	1.98E-08	-1.52475
P2RY1	9.47E-11	2E-08	-1.03323
CYP2B6	9.59E-11	2.01E-08	-1.33615
CTHRC1	9.67E-11	2.03E-08	1.610695
ARNTL2	1.02E-10	2.12E-08	1.330956
IFITM2	1.06E-10	2.2E-08	1.160021
GABRP	1.12E-10	2.28E-08	1.10729
HCAR3	1.18E-10	2.38E-08	1.769036
COL4A1	1.23E-10	2.46E-08	1.37192
PITX2	1.23E-10	2.47E-08	-1.72053
NPY1R	1.25E-10	2.49E-08	-1.18566
WDR78	1.3E-10	2.58E-08	-1.1375
AIFM3	1.38E-10	2.69E-08	-1.06092
XPNPEP2	1.38E-10	2.69E-08	-1.06134

CSF3R	1.41E-10	2.76E-08	1.11319
IFIT3	1.42E-10	2.76E-08	1.177642
CALCRL	1.48E-10	2.85E-08	1.132936
IFITM3	1.55E-10	2.94E-08	1.096485
PLEK	1.59E-10	3.01E-08	1.391337
SLC39A5	1.59E-10	3.01E-08	-1.05507
LRRC31	1.61E-10	3.03E-08	-1.05208
CD55	1.67E-10	3.13E-08	1.681124
SAA2	1.75E-10	3.26E-08	1.513783
BANK1	1.76E-10	3.27E-08	1.103298
CASP1	1.9E-10	3.47E-08	1.18814
UBE2L6	2.05E-10	3.67E-08	1.123236
LPCAT1	2.16E-10	3.86E-08	1.49496
ABCA12	2.29E-10	4.06E-08	1.645419
SLC35G1	2.3E-10	4.12E-08	-1.15194
KLHL6	2.52E-10	4.45E-08	1.018984
RAB31	2.77E-10	4.82E-08	1.222905
ANXA1	2.85E-10	4.94E-08	1.400833
ICOS	2.92E-10	5.04E-08	1.062862
TGM2	3E-10	5.15E-08	1.423564
SULF1	3.11E-10	5.31E-08	1.032504
LY96	3.22E-10	5.42E-08	1.025204
LYN	3.25E-10	5.44E-08	1.011068
THBD	3.52E-10	5.86E-08	1.038449
SOCS1	3.84E-10	6.26E-08	1.187095
PLA2G2A	3.89E-10	6.28E-08	1.406211
ENG	3.94E-10	6.33E-08	1.094378
ABCA8	3.91E-10	6.56E-08	-1.05484
CDC25B	4.3E-10	6.8E-08	1.15534
ENTPD1	4.34E-10	6.82E-08	1.038199
MGP	4.38E-10	6.85E-08	1.525082
ECSCR	4.52E-10	7.02E-08	1.076683
CTSH	4.92E-10	7.58E-08	1.007913
VLDLR	4.68E-10	7.59E-08	-1.47329
VAV3	4.68E-10	7.59E-08	-1.05726
CD274	5.07E-10	7.68E-08	1.542416
CRELD2	5.34E-10	7.99E-08	1.022433
PCDH17	5.35E-10	7.99E-08	1.220008
GPX8	5.5E-10	8.13E-08	1.301798
RAVER2	5.22E-10	8.25E-08	-1.07307
GPR183	5.95E-10	8.57E-08	1.073687
ZBP1	6.22E-10	8.9E-08	1.189089
FCGR3B	6.24E-10	8.9E-08	1.86833
VWF	6.28E-10	8.92E-08	1.353922
TMEM45A	6.34E-10	8.96E-08	1.038459
BGN	6.54E-10	9.16E-08	1.375213

PTGS2	6.56E-10	9.16E-08	1.320442
COL1A1	6.85E-10	9.52E-08	1.191116
CYP4X1	6.96E-10	9.58E-08	1.052416
CXCR4	7.17E-10	9.7E-08	1.129474
CHST15	7.17E-10	9.7E-08	1.010709
FOXQ1	7.26E-10	9.75E-08	1.305368
FDCSP	7.86E-10	1.05E-07	1.420469
HCLS1	8.85E-10	1.16E-07	1.008736
LRP8	8.98E-10	1.17E-07	1.178057
TMIGD1	8.83E-10	1.28E-07	-1.27533
NPY	8.98E-10	1.28E-07	-1.00958
ISG20	1.05E-09	1.34E-07	1.131657
SLC19A3	9.78E-10	1.38E-07	-1.06547
RAMP3	1.11E-09	1.4E-07	1.037228
SLCO1B3	1.13E-09	1.42E-07	1.360857
PXDN	1.16E-09	1.45E-07	1.02775
RGS5	1.16E-09	1.45E-07	1.396497
CASP5	1.17E-09	1.46E-07	1.046442
LILRB2	1.23E-09	1.51E-07	1.164262
DRAM1	1.23E-09	1.51E-07	1.023346
PLCD1	1.15E-09	1.57E-07	-1.03778
CXCR2	1.36E-09	1.64E-07	1.398923
PSMB9	1.37E-09	1.64E-07	1.037508
GNA15	1.39E-09	1.66E-07	1.026885
ACSL4	1.42E-09	1.68E-07	1.10135
PSAT1	1.46E-09	1.71E-07	1.315564
MXRA5	1.5E-09	1.75E-07	1.110535
VSIG1	1.52E-09	1.75E-07	1.523606
CD93	1.52E-09	1.75E-07	1.206539
IRAK3	1.63E-09	1.86E-07	1.133258
MS4A1	1.71E-09	1.92E-07	1.359478
ACKR4	1.71E-09	1.92E-07	1.096165
PRAP1	1.49E-09	1.95E-07	-1.41175
MSN	1.74E-09	1.95E-07	1.085235
TINAG	1.54E-09	1.99E-07	-1.01225
ICAM2	1.83E-09	2.03E-07	1.00193
RGCC	1.87E-09	2.06E-07	1.080455
CPXM1	1.88E-09	2.06E-07	1.021422
DEFB1	1.75E-09	2.2E-07	-1.4727
GHR	1.85E-09	2.31E-07	-1.32661
NID1	2.15E-09	2.31E-07	1.005781
COL5A2	2.21E-09	2.37E-07	1.009786
OSMR	2.27E-09	2.41E-07	1.425921
MIER3	1.96E-09	2.42E-07	-1.03346
LIPG	2.3E-09	2.43E-07	1.242997
TMEM72	2.05E-09	2.51E-07	-1.02332

PHLDA1	2.7E-09	2.81E-07	1.293048
C1S	2.7E-09	2.81E-07	1.086801
NAT8B	2.38E-09	2.82E-07	-1.10334
SRD5A3	2.79E-09	2.9E-07	1.074486
CD79A	2.87E-09	2.94E-07	1.291236
ART3	2.9E-09	2.97E-07	1.101826
CYP3A4	2.66E-09	3.06E-07	-1.08369
EPHX2	2.9E-09	3.27E-07	-1.12047
CA2	2.92E-09	3.28E-07	-1.01998
HSD3B2	3.05E-09	3.41E-07	-1.50431
HEG1	3.63E-09	3.61E-07	1.00784
FBN1	4.42E-09	4.28E-07	1.070119
COL1A2	4.57E-09	4.42E-07	1.067336
PNOC	4.65E-09	4.46E-07	1.028249
LRRN2	4.27E-09	4.46E-07	-1.1619
CNNM2	4.27E-09	4.46E-07	-1.0315
SUGCT	4.36E-09	4.52E-07	-1.00375
SERPINB3	5.04E-09	4.81E-07	1.578141
TGFBI	5.22E-09	4.94E-07	1.051734
CLDN2	5.41E-09	5.07E-07	1.531637
C2CD4A	5.47E-09	5.09E-07	1.646695
IL7R	5.53E-09	5.1E-07	1.29012
CHN1	5.69E-09	5.21E-07	1.082548
GCNT2	5.27E-09	5.35E-07	-1.01926
LYPD8	5.29E-09	5.35E-07	-1.1026
RSPO3	5.97E-09	5.4E-07	1.004925
SDR16C5	6.36E-09	5.72E-07	1.09433
CCL2	6.44E-09	5.77E-07	1.249774
DDIT4	7.1E-09	6.31E-07	1.15649
IL24	7.18E-09	6.35E-07	1.181679
RPS6KA6	6.89E-09	6.68E-07	-1.1595
TRIB2	7.98E-09	6.94E-07	1.293983
PRKG2	7.62E-09	7.32E-07	-1.23163
IL1A	8.64E-09	7.48E-07	1.066783
SERPING1	8.98E-09	7.73E-07	1.133791
DSG3	9.39E-09	8.01E-07	1.03385
COL6A3	9.45E-09	8.04E-07	1.308154
CDH5	9.7E-09	8.24E-07	1.019272
FGR	1.03E-08	8.64E-07	1.056333
IFI44	1.05E-08	8.77E-07	1.009775
BCL2A1	1.07E-08	8.9E-07	1.492737
SLC7A11	1.15E-08	9.4E-07	1.2588
CARD6	1.16E-08	9.5E-07	1.102464
MT1H	1.06E-08	9.5E-07	-1.1021
KLHL5	1.17E-08	9.51E-07	1.090972
TMEM158	1.19E-08	9.67E-07	1.205555

KRT6B	1.24E-08	9.94E-07	1.426257
SLC38A4	1.26E-08	1.08E-06	-1.44267
TNFAIP6	1.44E-08	1.12E-06	1.264748
THRB	1.44E-08	1.19E-06	-1.14423
ALDOB	1.56E-08	1.19E-06	1.446892
CNTFR	1.49E-08	1.22E-06	-1.2036
FFAR2	1.65E-08	1.24E-06	1.028385
TRHDE	1.66E-08	1.33E-06	-1.31429
SLC16A14	2.23E-08	1.57E-06	1.040851
CYP2B7P	2.34E-08	1.74E-06	-1.2153
CCDC3	2.64E-08	1.82E-06	1.195398
CEMIP	2.73E-08	1.88E-06	1.612039
TRIM40	2.77E-08	1.9E-06	1.072745
COL15A1	2.91E-08	1.98E-06	1.107755
GBP1	2.92E-08	1.99E-06	1.06812
DEFB4A	2.97E-08	2.02E-06	1.323086
ROBO1	3.18E-08	2.12E-06	1.060355
SLC51B	3.14E-08	2.24E-06	-1.13564
S100A7	3.59E-08	2.37E-06	1.016315
PDE4B	4.16E-08	2.67E-06	1.064747
THEMIS2	4.39E-08	2.78E-06	1.053434
STOM	4.65E-08	2.91E-06	1.007609
STEAP4	4.65E-08	2.91E-06	1.288392
ME1	5.25E-08	3.23E-06	1.058262
CHST2	5.31E-08	3.26E-06	1.023512
SLC51A	5.44E-08	3.45E-06	-1.53764
BRINP3	5.44E-08	3.45E-06	-1.13585
CTLA4	6.32E-08	3.78E-06	1.031564
SLC37A2	6.17E-08	3.84E-06	-1.04653
SCD	6.91E-08	4.03E-06	1.038455
CCL21	7E-08	4.06E-06	1.01714
SLC23A1	6.92E-08	4.21E-06	-1.08102
AGT	7.49E-08	4.31E-06	1.138227
PITPNC1	7.62E-08	4.36E-06	1.138061
EGFL6	8.25E-08	4.64E-06	1.152643
B4GALNT2	8.86E-08	5.22E-06	-1.25605
SCNN1B	9.48E-08	5.47E-06	-1.16785
NXPE4	9.78E-08	5.61E-06	-1.12493
TEX11	1.21E-07	6.7E-06	-1.04295
ELOVL5	1.32E-07	6.82E-06	1.070836
CSF2RB	1.45E-07	7.39E-06	1.146437
TFPI2	1.82E-07	8.81E-06	1.121042
SLC7A5	2.03E-07	9.56E-06	1.232378
SAA1	2.14E-07	9.99E-06	1.314376
COL12A1	2.15E-07	9.99E-06	1.062102
CCL24	2.42E-07	1.1E-05	1.122949

F2RL2	2.54E-07	1.13E-05	1.01831
CCL4	2.64E-07	1.17E-05	1.083003
RGS1	2.79E-07	1.22E-05	1.084681
NPTX2	2.84E-07	1.24E-05	1.043917
SLC13A2	2.63E-07	1.27E-05	-1.18275
G0S2	3.72E-07	1.55E-05	1.001391
INHBA	3.78E-07	1.57E-05	1.015373
OAS2	4.76E-07	1.9E-05	1.03551
DPP10-AS1	4.33E-07	1.92E-05	-1.19848
TWIST1	5.05E-07	1.99E-05	1.043816
OTOP2	5.31E-07	2.25E-05	-1.12805
HSPB3	6.2E-07	2.55E-05	-1.20405
LAMA1	7.64E-07	3.04E-05	-1.09159
APOBEC3B	8E-07	3.16E-05	-1.12981
SFRP2	9.35E-07	3.29E-05	1.105724
SERPINE1	1.17E-06	3.95E-05	1.182733
LOXL2	2.03E-06	6.27E-05	1.090167
KCND3	3.64E-06	0.000101	1.275163
PI15	4.16E-06	0.000112	1.096194
WNT5A	6.06E-06	0.000154	1.046116
PFKFB3	6.43E-06	0.000162	1.028346

Table S2: Fibroblast-related differential genes in the ScRAN dataset.

Cluster	Gene	Avg_log2FC	Pct.1	*p*_val_adj
Fibroblasts	CLTC	0.250758	0.316	0.009748
Fibroblasts	H3F3A	0.262997	0.805	1.12E-23
Fibroblasts	JMJD1C	0.291192	0.366	0.002335
Fibroblasts	MTRNR2L12	0.293824	0.771	1.3E-13
Fibroblasts	GBP2	0.299469	0.303	2.97E-27
Fibroblasts	HSPH1	0.299988	0.264	0.000816
Fibroblasts	SQOR	0.301203	0.26	0.000567
Fibroblasts	SERINC1	0.312044	0.314	2.85E-05
Fibroblasts	ISG15	0.314161	0.394	1.16E-17
Fibroblasts	RPLP1	0.317495	0.9	5.1E-15
Fibroblasts	LRP10	0.326028	0.333	0.000232
Fibroblasts	NINJ1	0.328009	0.307	3.84E-19
Fibroblasts	CAPZA2	0.339205	0.331	0.005966
Fibroblasts	RAB14	0.340974	0.258	0.001088
Fibroblasts	LAMP1	0.343905	0.258	2.35E-24
Fibroblasts	ACTR10	0.345509	0.258	0.000337
Fibroblasts	TACC1	0.353285	0.262	0.000173
Fibroblasts	LIMS1	0.362943	0.413	9.49E-21
Fibroblasts	GNB2	0.36899	0.281	0.000105

Fibroblasts	C14orf119	0.369623	0.268	0.003502
Fibroblasts	IFT57	0.371947	0.266	1.73E-05
Fibroblasts	MIER1	0.372436	0.277	0.000695
Fibroblasts	KLF6	0.37493	0.6	1.45E-05
Fibroblasts	DYNC1H1	0.376168	0.312	0.000115
Fibroblasts	H2AFY	0.3765	0.329	3.13E-15
Fibroblasts	RBPJ	0.376656	0.331	2.03E-05
Fibroblasts	CCT6A	0.377993	0.292	0.00059
Fibroblasts	BAX	0.379016	0.329	4.05E-05
Fibroblasts	CHD4	0.380174	0.268	0.000336
Fibroblasts	RPL21	0.38141	0.773	0.000153
Fibroblasts	COA4	0.381623	0.31	0.002091
Fibroblasts	EIF1	0.382833	0.874	2.01E-20
Fibroblasts	SETD5	0.389134	0.258	5.68E-05
Fibroblasts	BTG1	0.389165	0.597	1.52E-14
Fibroblasts	RPL37A	0.389995	0.74	0.001954
Fibroblasts	PRDX1	0.397387	0.621	0.005974
Fibroblasts	DAZAP2	0.399397	0.383	3.69E-05
Fibroblasts	CTDNEP1	0.402068	0.288	0.001083
Fibroblasts	CALCOCO2	0.405905	0.305	0.008819
Fibroblasts	PLSCR1	0.407169	0.333	6.74E-43
Fibroblasts	DNAJB14	0.409303	0.264	4.97E-05
Fibroblasts	SMARCA5	0.411396	0.26	0.000698
Fibroblasts	GABARAPL1	0.413543	0.275	6.96E-18
Fibroblasts	NR3C1	0.416848	0.318	7.05E-06
Fibroblasts	SREK1	0.418543	0.29	0.008091
Fibroblasts	CAPN2	0.418623	0.284	1.27E-13
Fibroblasts	EIF4E2	0.418771	0.323	1.48E-05
Fibroblasts	UBE2I	0.419191	0.359	0.001543
Fibroblasts	SYF2	0.419961	0.394	0.003928
Fibroblasts	RPS15A	0.422444	0.848	1.56E-20
Fibroblasts	BORCS7	0.42303	0.251	0.000274
Fibroblasts	SPPL2A	0.426599	0.266	2.43E-16
Fibroblasts	VDAC3	0.427196	0.323	0.000355
Fibroblasts	RAB5C	0.434371	0.374	9.54E-13
Fibroblasts	RSF1	0.434535	0.303	0.000135
Fibroblasts	STAT1	0.435036	0.29	0.000534
Fibroblasts	BRD4	0.436746	0.29	1.6E-05
Fibroblasts	SELENOP	0.43897	0.355	1.5E-184
Fibroblasts	ARID1B	0.441113	0.314	0.000121
Fibroblasts	COX6B1	0.44194	0.652	0.000282
Fibroblasts	NMT1	0.442102	0.262	2.24E-05
Fibroblasts	PTEN	0.445151	0.29	2.41E-25
Fibroblasts	PAK2	0.44585	0.294	1.35E-05
Fibroblasts	PARP14	0.448656	0.273	0.002642
Fibroblasts	MRPL27	0.449306	0.31	8.58E-05

Fibroblasts	HMGN2	0.450525	0.42	0.004198
Fibroblasts	TMEM30A	0.450634	0.281	5.07E-14
Fibroblasts	NDUFS4	0.453462	0.325	0.000417
Fibroblasts	PABPC1	0.454968	0.595	6.77E-05
Fibroblasts	CDC42SE1	0.456199	0.297	9.67E-05
Fibroblasts	RPL27	0.45729	0.68	0.000196
Fibroblasts	BLVRB	0.463508	0.359	0.000125
Fibroblasts	CCT2	0.464235	0.329	0.000346
Fibroblasts	PPP1R12A	0.465875	0.338	1.25E-05
Fibroblasts	RPL29	0.466766	0.812	4.47E-19
Fibroblasts	PLIN3	0.469512	0.279	5.07E-13
Fibroblasts	LARP7	0.470947	0.299	0.00019
Fibroblasts	RTF1	0.476647	0.325	1.85E-05
Fibroblasts	MARK3	0.477884	0.266	0.000274
Fibroblasts	LUZP1	0.478046	0.268	3.07E-39
Fibroblasts	RPS3	0.47918	0.794	1.45E-12
Fibroblasts	RPL35	0.479496	0.799	1.85E-18
Fibroblasts	PRKAR1A	0.480223	0.379	2.06E-13
Fibroblasts	AP3S1	0.48025	0.31	0.000171
Fibroblasts	ATP5F1E	0.481347	0.812	1.34E-16
Fibroblasts	ECE1	0.482716	0.268	5.29E-22
Fibroblasts	POLR2F	0.492387	0.361	1.97E-05
Fibroblasts	FAU	0.494795	0.883	1.34E-26
Fibroblasts	CTDSP2	0.496918	0.26	1.18E-21
Fibroblasts	RAN	0.497771	0.55	0.003372
Fibroblasts	METTL26	0.497924	0.26	9.4E-06
Fibroblasts	CMIP	0.497979	0.281	4.6E-42
Fibroblasts	EIF4G2	0.499302	0.519	0.000435
Fibroblasts	NFAT5	0.500503	0.275	5.14E-20
Fibroblasts	RPS14	0.501914	0.848	1.4E-24
Fibroblasts	CHMP3	0.501977	0.307	1.01E-22
Fibroblasts	ASAP1	0.502522	0.255	1.47E-36
Fibroblasts	POLR2G	0.505121	0.284	8.18E-14
Fibroblasts	LAP3	0.518581	0.422	0.00908
Fibroblasts	TSPO	0.518689	0.392	8.05E-06
Fibroblasts	VEGFA	0.518697	0.286	1.42E-27
Fibroblasts	UBA52	0.519675	0.771	1.09E-17
Fibroblasts	KMT2A	0.52005	0.307	8.66E-05
Fibroblasts	SH3BGRL	0.522001	0.45	0.000259
Fibroblasts	TROVE2	0.522042	0.275	0.000279
Fibroblasts	TNFSF10	0.5224	0.323	3.88E-34
Fibroblasts	UBL5	0.522418	0.654	7.56E-05
Fibroblasts	COMMD4	0.527893	0.275	0.00402
Fibroblasts	FBH1	0.528813	0.258	0.003869
Fibroblasts	SLC39A1	0.528971	0.31	6.51E-36
Fibroblasts	RPS15	0.530049	0.812	4.26E-14

Fibroblasts	MVP	0.533451	0.32	2.07E-16
Fibroblasts	NCL	0.534636	0.58	0.00598
Fibroblasts	PCM1	0.535605	0.299	3.03E-17
Fibroblasts	ANXA11	0.537369	0.357	4.22E-20
Fibroblasts	ITGB1BP1	0.538407	0.279	4.23E-29
Fibroblasts	GADD45GIP1	0.538689	0.316	0.000956
Fibroblasts	SRSF9	0.53974	0.348	0.004934
Fibroblasts	MAP1LC3B	0.541061	0.392	0.001128
Fibroblasts	GSTO1	0.541191	0.502	1.18E-14
Fibroblasts	COX20	0.543775	0.32	2.28E-13
Fibroblasts	ATP5MC3	0.548893	0.619	0.00064
Fibroblasts	ETS1	0.550597	0.255	1.77E-34
Fibroblasts	ENG	0.551133	0.288	4.1E-115
Fibroblasts	CRTAP	0.553358	0.288	2.08E-36
Fibroblasts	MFSD10	0.554056	0.251	2.34E-39
Fibroblasts	PRDX5	0.558292	0.582	0.000453
Fibroblasts	RPS21	0.558339	0.792	5.63E-22
Fibroblasts	ZNF428	0.560671	0.26	3.65E-18
Fibroblasts	COX8A	0.561949	0.569	0.002329
Fibroblasts	MCFD2	0.562392	0.307	0.001138
Fibroblasts	CHURC1	0.563331	0.368	1.25E-18
Fibroblasts	STUB1	0.563662	0.286	1.89E-05
Fibroblasts	RBM39	0.564057	0.619	0.000188
Fibroblasts	YWHAB	0.565835	0.602	4.75E-14
Fibroblasts	PIN4	0.568959	0.303	0.007772
Fibroblasts	PSMA4	0.569679	0.413	0.002572
Fibroblasts	CAST	0.569959	0.433	1.17E-14
Fibroblasts	PBDC1	0.572286	0.281	5.33E-17
Fibroblasts	VPS29	0.572512	0.461	1.02E-05
Fibroblasts	CLTB	0.573437	0.277	4.51E-21
Fibroblasts	ABHD5	0.574838	0.264	2.05E-14
Fibroblasts	CTNNA1	0.575083	0.301	5.95E-28
Fibroblasts	TXNL1	0.576327	0.448	0.002363
Fibroblasts	ZFAND5	0.5765	0.355	1.18E-12
Fibroblasts	IFITM2	0.576877	0.723	5.4E-145
Fibroblasts	SHC1	0.577824	0.251	8.62E-22
Fibroblasts	TIMM13	0.57822	0.359	1.95E-05
Fibroblasts	AHCYL1	0.579085	0.294	1.04E-22
Fibroblasts	C12orf10	0.580107	0.279	3.05E-16
Fibroblasts	CHD9	0.581018	0.353	4.42E-05
Fibroblasts	GTF2H5	0.581326	0.266	5.33E-16
Fibroblasts	SDHD	0.581938	0.344	0.001311
Fibroblasts	ZCCHC17	0.582415	0.316	5.32E-21
Fibroblasts	RPS27	0.583331	0.842	5.43E-22
Fibroblasts	PTS	0.585079	0.303	5.44E-19
Fibroblasts	BAD	0.585926	0.273	1.33E-12

Fibroblasts	PGAM1	0.587584	0.396	0.000527
Fibroblasts	GUK1	0.589011	0.535	0.00793
Fibroblasts	MT-ND3	0.590233	0.978	5.39E-24
Fibroblasts	MT-CO2	0.593135	0.981	2.04E-27
Fibroblasts	SMIM7	0.594273	0.392	0.000822
Fibroblasts	REV3L	0.598267	0.255	2.47E-30
Fibroblasts	AES	0.599911	0.364	1.58E-20
Fibroblasts	CIRBP	0.600212	0.574	0.005272
Fibroblasts	PCBP2	0.600783	0.478	0.002506
Fibroblasts	CSGALNACT2	0.601936	0.271	1.2E-23
Fibroblasts	ZEB1	0.603144	0.255	2.06E-26
Fibroblasts	ZFAS1	0.603798	0.455	5.93E-14
Fibroblasts	CCDC85B	0.604022	0.255	1.18E-12
Fibroblasts	PRDX6	0.60421	0.468	0.000227
Fibroblasts	RPL41	0.604474	0.931	6.22E-44
Fibroblasts	WDR83OS	0.605035	0.526	0.000637
Fibroblasts	MPZL1	0.606354	0.268	2.32E-30
Fibroblasts	HNRNPA0	0.606535	0.331	2.28E-14
Fibroblasts	TUBB4B	0.607254	0.396	0.000288
Fibroblasts	SNRPG	0.60898	0.528	0.003801
Fibroblasts	CAPNS1	0.609985	0.346	9.46E-30
Fibroblasts	C1orf123	0.612612	0.301	1.55E-33
Fibroblasts	RCN2	0.614452	0.251	3.74E-35
Fibroblasts	MYO1C	0.615011	0.305	2.97E-46
Fibroblasts	BZW1	0.615219	0.457	1.06E-13
Fibroblasts	HNRNPA1	0.616147	0.552	0.000191
Fibroblasts	PDE4DIP	0.616282	0.288	7.84E-27
Fibroblasts	OAT	0.61782	0.281	2.56E-21
Fibroblasts	PAFAH1B1	0.618003	0.29	0.000271
Fibroblasts	COX4I1	0.618648	0.703	1.01E-13
Fibroblasts	PFDN5	0.618874	0.71	8.12E-15
Fibroblasts	LDLR	0.619735	0.253	3.62E-22
Fibroblasts	HACD3	0.622153	0.268	2.17E-19
Fibroblasts	CTSL	0.622767	0.422	1.6E-155
Fibroblasts	PRNP	0.624954	0.318	3.84E-35
Fibroblasts	TCEAL8	0.625042	0.353	4.91E-16
Fibroblasts	BRI3	0.625718	0.264	1.19E-18
Fibroblasts	RPL18	0.626646	0.857	5.18E-36
Fibroblasts	JAK1	0.626699	0.426	2.6E-18
Fibroblasts	IAH1	0.626992	0.351	4.55E-16
Fibroblasts	CTSA	0.629381	0.366	7.67E-30
Fibroblasts	S100A16	0.630093	0.299	2.8E-186
Fibroblasts	DYNLT3	0.630292	0.299	2.22E-30
Fibroblasts	HIF1A	0.630347	0.474	2.49E-37
Fibroblasts	ANXA6	0.630643	0.351	0.001657
Fibroblasts	RPS6	0.630963	0.784	5.39E-16

Fibroblasts	HMGN1	0.632013	0.381	0.002944
Fibroblasts	ICAM1	0.633473	0.34	4.8E-27
Fibroblasts	SRSF6	0.634748	0.264	0.000277
Fibroblasts	GLT8D2	0.635177	0.271	0
Fibroblasts	CBX5	0.636452	0.297	1.56E-12
Fibroblasts	RCN1	0.636745	0.288	3.99E-37
Fibroblasts	SPTAN1	0.63703	0.281	1.74E-22
Fibroblasts	STMP1	0.637141	0.374	2.49E-17
Fibroblasts	CBX3	0.637422	0.431	5.73E-14
Fibroblasts	SIVA1	0.637891	0.403	0.00124
Fibroblasts	NDUFB8	0.638197	0.556	0.005238
Fibroblasts	TNFAIP3	0.639696	0.426	1.29E-12
Fibroblasts	NOP53	0.639937	0.275	0.000288
Fibroblasts	CBR1	0.641084	0.271	2.69E-23
Fibroblasts	PNRC2	0.64249	0.372	1.56E-26
Fibroblasts	YME1L1	0.644285	0.353	0.005486
Fibroblasts	DEGS1	0.645805	0.329	1.34E-19
Fibroblasts	NDUFB9	0.646985	0.435	9.57E-05
Fibroblasts	PHPT1	0.647231	0.576	0.0001
Fibroblasts	SRP9	0.648662	0.446	7.16E-05
Fibroblasts	IFI16	0.650606	0.435	1.16E-15
Fibroblasts	ADD1	0.65086	0.29	2.75E-16
Fibroblasts	VAMP5	0.650914	0.411	2.17E-14
Fibroblasts	NORAD	0.652115	0.338	4.84E-24
Fibroblasts	ELF2	0.653391	0.288	3.22E-20
Fibroblasts	PSMG2	0.656354	0.394	3.61E-14
Fibroblasts	SNHG7	0.65656	0.255	1.43E-19
Fibroblasts	COX5B	0.657424	0.558	0.002537
Fibroblasts	PPIB	0.658569	0.773	5.5E-22
Fibroblasts	PIM3	0.6605	0.281	0.000641
Fibroblasts	RBFOX2	0.661275	0.273	1.9E-238
Fibroblasts	MAP3K20	0.662023	0.297	4.7E-117
Fibroblasts	PYURF	0.662894	0.297	6.89E-22
Fibroblasts	RPL7A	0.663008	0.872	6.35E-44
Fibroblasts	MPG	0.663739	0.329	0.000593
Fibroblasts	GZMK	0.664492	0.385	1.47E-05
Fibroblasts	UQCR11	0.664538	0.593	0.001218
Fibroblasts	HEBP2	0.669057	0.273	1.13E-22
Fibroblasts	TBCB	0.669898	0.292	1.29E-34
Fibroblasts	MT-ND5	0.671083	0.872	1.21E-29
Fibroblasts	MAGED2	0.671407	0.359	0.001885
Fibroblasts	DESI2	0.672187	0.268	5.78E-46
Fibroblasts	MRPL41	0.675347	0.303	0.000378
Fibroblasts	C1orf21	0.677891	0.255	8.9E-203
Fibroblasts	TJP1	0.681275	0.253	2E-190
Fibroblasts	SCAND1	0.682009	0.314	0.001655

Fibroblasts	ATP6V0E1	0.682134	0.641	4.94E-16
Fibroblasts	BDH2	0.68289	0.303	6.5E-130
Fibroblasts	EIF5A	0.684709	0.485	9.64E-05
Fibroblasts	WSB1	0.686499	0.5	4.62E-23
Fibroblasts	GPX8	0.687002	0.268	0
Fibroblasts	ATP5MF	0.687196	0.582	0.000747
Fibroblasts	CBX6	0.687291	0.303	2.67E-35
Fibroblasts	SRSF11	0.690142	0.476	2.88E-13
Fibroblasts	WARS	0.691231	0.424	6.9E-27
Fibroblasts	TRIR	0.692204	0.392	1.72E-05
Fibroblasts	REEP3	0.693525	0.264	2.4E-37
Fibroblasts	CCL4	0.69763	0.909	1.81E-50
Fibroblasts	RHOBTB3	0.699194	0.284	1.1E-260
Fibroblasts	ANXA1	0.700162	0.543	2.6E-121
Fibroblasts	RPL8	0.700617	0.84	1.2E-24
Fibroblasts	RPL10	0.70086	0.935	4.48E-48
Fibroblasts	CNOT4	0.703562	0.277	0.000162
Fibroblasts	ACTN4	0.704536	0.424	3.45E-14
Fibroblasts	FKBP1A	0.704938	0.45	3.12E-16
Fibroblasts	FNIP1	0.706487	0.284	2.7E-23
Fibroblasts	RPL31	0.70689	0.647	1.3E-12
Fibroblasts	MTCH1	0.707327	0.34	4.27E-44
Fibroblasts	MT-ND4	0.708914	0.97	6.02E-31
Fibroblasts	ARL6IP4	0.712617	0.457	0.00385
Fibroblasts	RPL36	0.716676	0.835	2.02E-38
Fibroblasts	AHNAK	0.716749	0.504	3.21E-16
Fibroblasts	BHLHE40	0.717752	0.318	5.31E-35
Fibroblasts	PARVA	0.718888	0.273	0
Fibroblasts	WWTR1	0.719126	0.26	3.7E-234
Fibroblasts	AKR7A2	0.720226	0.292	3.26E-47
Fibroblasts	PRPF4B	0.720647	0.377	0.00423
Fibroblasts	AFF4	0.723653	0.335	6.8E-22
Fibroblasts	RSRP1	0.724239	0.478	1.03E-05
Fibroblasts	COX7C	0.725077	0.699	4.23E-14
Fibroblasts	LAMTOR4	0.726844	0.565	1.25E-12
Fibroblasts	DDAH2	0.726973	0.325	7.5E-38
Fibroblasts	CSNK1A1	0.727513	0.455	2.81E-14
Fibroblasts	ARL6IP5	0.727922	0.491	8.34E-19
Fibroblasts	MT-ND2	0.72885	0.942	2.94E-49
Fibroblasts	ALDH2	0.728985	0.314	1.54E-44
Fibroblasts	AP2S1	0.729344	0.487	6.01E-13
Fibroblasts	MIDN	0.729426	0.409	2.54E-34
Fibroblasts	TMEM165	0.730536	0.351	5.63E-23
Fibroblasts	PHF14	0.732377	0.268	1.5E-39
Fibroblasts	GSTP1	0.733531	0.762	1.94E-22
Fibroblasts	RPL24	0.735226	0.801	2.6E-34

Fibroblasts	PPP3CA	0.736561	0.323	2.35E-20
Fibroblasts	NDFIP1	0.736815	0.342	2.33E-15
Fibroblasts	RPS28	0.743088	0.89	1.83E-45
Fibroblasts	DDT	0.743372	0.42	0.000213
Fibroblasts	RNH1	0.744024	0.418	1.08E-12
Fibroblasts	PFDN2	0.746704	0.472	2.08E-14
Fibroblasts	GABARAPL2	0.749831	0.474	3.52E-12
Fibroblasts	DYNLT1	0.750549	0.496	2.29E-22
Fibroblasts	ISG20	0.752985	0.344	1.72E-52
Fibroblasts	PPFIBP1	0.753713	0.303	8E-217
Fibroblasts	TMEM98	0.754425	0.286	0
Fibroblasts	ARF5	0.75678	0.314	8.77E-46
Fibroblasts	YBX1	0.758782	0.571	1.12E-21
Fibroblasts	C8orf59	0.758857	0.541	2.44E-05
Fibroblasts	FAAP20	0.763203	0.279	8.38E-21
Fibroblasts	CARHSP1	0.768946	0.405	8.76E-22
Fibroblasts	RPL19	0.76939	0.844	3.71E-42
Fibroblasts	ELOC	0.769917	0.437	3.74E-05
Fibroblasts	MACF1	0.770204	0.379	2.56E-27
Fibroblasts	ID2	0.771031	0.294	2.42E-30
Fibroblasts	NACA	0.772591	0.81	1.41E-37
Fibroblasts	SIGIRR	0.772736	0.327	5.25E-38
Fibroblasts	SSB	0.774084	0.474	3.49E-23
Fibroblasts	PTGR1	0.780579	0.31	0
Fibroblasts	BNIP3L	0.780934	0.411	1.01E-28
Fibroblasts	ANKRD10	0.781456	0.31	3.58E-36
Fibroblasts	MZT2B	0.782066	0.418	4.6E-05
Fibroblasts	ATP5F1D	0.782506	0.409	1.57E-13
Fibroblasts	PDLIM2	0.783098	0.312	2.4E-228
Fibroblasts	VKORC1	0.785621	0.426	1.2E-05
Fibroblasts	ENAH	0.785656	0.26	0
Fibroblasts	NEDD8	0.785672	0.587	7.8E-15
Fibroblasts	RPL10A	0.787559	0.734	3.78E-17
Fibroblasts	FGFR1	0.788006	0.284	1.5E-276
Fibroblasts	PDIA3	0.789384	0.53	2.04E-12
Fibroblasts	PMEPA1	0.789511	0.279	1E-26
Fibroblasts	SNX6	0.790677	0.437	1.85E-29
Fibroblasts	ATP6AP2	0.792008	0.433	6.55E-16
Fibroblasts	MRFAP1	0.792268	0.465	1.44E-20
Fibroblasts	FBLIM1	0.793381	0.314	9.1E-250
Fibroblasts	HEBP1	0.793642	0.281	1.1E-165
Fibroblasts	SCARB2	0.793811	0.392	1.02E-30
Fibroblasts	CISD1	0.794814	0.34	4.71E-35
Fibroblasts	COMT	0.795023	0.418	1.63E-31
Fibroblasts	TUBA1C	0.798647	0.342	1E-26
Fibroblasts	HSP90AA1	0.799422	0.729	3.46E-16

Fibroblasts	C4orf48	0.800589	0.277	1.63E-38
Fibroblasts	P4HA2	0.801232	0.294	1.8E-171
Fibroblasts	GRK5	0.802074	0.284	2.7E-123
Fibroblasts	APLP2	0.802579	0.392	4.46E-47
Fibroblasts	TXN	0.803666	0.671	2.88E-23
Fibroblasts	DUT	0.804457	0.331	3.27E-41
Fibroblasts	ANTXR1	0.804684	0.286	0
Fibroblasts	CSF1	0.804942	0.279	3.3E-214
Fibroblasts	RBM17	0.805811	0.331	1.33E-15
Fibroblasts	C1orf56	0.806749	0.281	9.12E-19
Fibroblasts	CNIH4	0.810112	0.387	5.55E-32
Fibroblasts	CYB5A	0.813967	0.366	1.73E-30
Fibroblasts	RPL7	0.814398	0.734	1.72E-25
Fibroblasts	SGCE	0.815561	0.31	4.6E-239
Fibroblasts	NECTIN2	0.815572	0.335	3.9E-140
Fibroblasts	HNRNPA2B1	0.816646	0.719	1.38E-23
Fibroblasts	HLA-A	0.816812	0.747	3.37E-21
Fibroblasts	MT-ND1	0.820003	0.922	3.82E-20
Fibroblasts	YWHAQ	0.820976	0.411	9.11E-22
Fibroblasts	RPLP0	0.823025	0.755	2.43E-25
Fibroblasts	EMP1	0.823717	0.31	2.8E-143
Fibroblasts	MT-ATP6	0.825936	0.972	1.34E-16
Fibroblasts	RPS3A	0.826003	0.842	9.68E-45
Fibroblasts	GNG12	0.829074	0.273	9.3E-267
Fibroblasts	SEPT2	0.82957	0.489	1.14E-15
Fibroblasts	ARL3	0.831091	0.392	1.41E-28
Fibroblasts	SAP18	0.83259	0.652	2.38E-19
Fibroblasts	UQCRQ	0.834948	0.68	2.92E-13
Fibroblasts	RPL13	0.836974	0.803	2.63E-16
Fibroblasts	RPL37	0.842318	0.851	1.16E-49
Fibroblasts	MSN	0.843191	0.461	5.54E-52
Fibroblasts	CTTN	0.845588	0.333	3.1E-164
Fibroblasts	TLN1	0.847296	0.407	3.08E-30
Fibroblasts	ERH	0.847633	0.567	1.06E-14
Fibroblasts	TMEM50A	0.852575	0.582	1.36E-20
Fibroblasts	DYNC1I2	0.853367	0.374	1.69E-34
Fibroblasts	GJA1	0.854774	0.268	1.6E-294
Fibroblasts	NDN	0.857505	0.305	0
Fibroblasts	PSMB9	0.859536	0.846	4.94E-52
Fibroblasts	PARK7	0.864488	0.654	2.6E-18
Fibroblasts	ST13	0.866144	0.485	3.12E-19
Fibroblasts	CLTA	0.867297	0.422	1.08E-32
Fibroblasts	ACTN1	0.868981	0.377	2.3E-140
Fibroblasts	NEXN	0.870367	0.262	0
Fibroblasts	FCGRT	0.870912	0.476	1.93E-28
Fibroblasts	ELOB	0.871395	0.738	1.99E-31

Fibroblasts	RPL28	0.872549	0.885	6.24E-54
Fibroblasts	PNRC1	0.873158	0.61	3.51E-27
Fibroblasts	RRBP1	0.875807	0.61	9.26E-14
Fibroblasts	BANF1	0.876388	0.433	2.05E-19
Fibroblasts	CRIP2	0.876514	0.292	9.2E-266
Fibroblasts	FKBP10	0.876884	0.281	0
Fibroblasts	PSMA7	0.877131	0.606	1.25E-18
Fibroblasts	ACSL4	0.878301	0.31	1.07E-34
Fibroblasts	AP2M1	0.88059	0.444	2.33E-15
Fibroblasts	BSG	0.881709	0.522	3.91E-13
Fibroblasts	SLC9A3R2	0.884237	0.305	1.6E-253
Fibroblasts	GSTM3	0.884471	0.348	1.1E-208
Fibroblasts	CXCR4	0.88563	0.305	1.7E-53
Fibroblasts	TGM2	0.886185	0.277	1.2E-158
Fibroblasts	UBE2E3	0.886332	0.372	2.77E-39
Fibroblasts	CCNI	0.88763	0.595	2.46E-24
Fibroblasts	SRSF10	0.889288	0.459	6.89E-17
Fibroblasts	CDC42	0.889336	0.658	4.48E-24
Fibroblasts	TMA7	0.890523	0.773	1.4E-33
Fibroblasts	SRSF3	0.891265	0.615	1.8E-15
Fibroblasts	NME3	0.89363	0.372	4.21E-15
Fibroblasts	ACTG1	0.893896	0.766	2.71E-32
Fibroblasts	SNRPD2	0.894079	0.532	1.07E-12
Fibroblasts	FAM133B	0.898766	0.481	4.73E-15
Fibroblasts	LAMB2	0.900137	0.294	0
Fibroblasts	CTNNB1	0.902244	0.459	1.25E-44
Fibroblasts	ETS2	0.90266	0.472	2E-135
Fibroblasts	PRRX1	0.902878	0.297	0
Fibroblasts	MYADM	0.903125	0.429	6.34E-17
Fibroblasts	RBMS3	0.904151	0.288	0
Fibroblasts	MT-ND4L	0.904281	0.693	2.48E-13
Fibroblasts	NDUFB1	0.906077	0.587	6.7E-17
Fibroblasts	CCNL1	0.91066	0.567	1.12E-14
Fibroblasts	MMP14	0.912404	0.29	8.6E-178
Fibroblasts	DDIT4	0.914299	0.859	1.66E-51
Fibroblasts	MRC2	0.914647	0.307	0
Fibroblasts	TBCA	0.918492	0.563	1.13E-14
Fibroblasts	KANK2	0.918699	0.294	0
Fibroblasts	KLF9	0.91896	0.305	2.4E-164
Fibroblasts	OLFML3	0.922162	0.318	0
Fibroblasts	SLC25A37	0.923181	0.47	1.5E-111
Fibroblasts	SMIM37	0.925729	0.333	3.96E-31
Fibroblasts	CLEC11A	0.927817	0.281	0
Fibroblasts	PLOD2	0.929435	0.279	0
Fibroblasts	KTN1	0.929638	0.5	6.6E-28
Fibroblasts	APP	0.929935	0.4	5.9E-223

Fibroblasts	PHACTR2	0.932417	0.361	1E-135
Fibroblasts	CALM1	0.934897	0.673	1.12E-19
Fibroblasts	DNAJB4	0.939284	0.292	2.24E-36
Fibroblasts	H2AFJ	0.939372	0.403	4.98E-23
Fibroblasts	RAB34	0.941512	0.359	1.2E-164
Fibroblasts	FAM20C	0.942255	0.294	0
Fibroblasts	WASF2	0.942626	0.468	1.54E-47
Fibroblasts	RARRES3	0.942661	0.478	6.59E-33
Fibroblasts	PBX1	0.943421	0.299	0
Fibroblasts	HP1BP3	0.94426	0.465	1.58E-19
Fibroblasts	GNG5	0.945912	0.617	1.31E-12
Fibroblasts	EVA1B	0.948512	0.318	1.1E-127
Fibroblasts	WTAP	0.94887	0.455	1.55E-25
Fibroblasts	NENF	0.949106	0.318	1.57E-29
Fibroblasts	C11orf58	0.95332	0.582	4.28E-20
Fibroblasts	NFE2L2	0.95471	0.539	2.74E-46
Fibroblasts	DKK3	0.957397	0.303	0
Fibroblasts	TPM4	0.958565	0.639	1.99E-34
Fibroblasts	WLS	0.960681	0.34	0
Fibroblasts	GGT5	0.965615	0.301	1.9E-273
Fibroblasts	EEF1A1	0.966747	0.909	5.67E-49
Fibroblasts	EHD2	0.967072	0.303	0
Fibroblasts	SPATS2L	0.970186	0.342	5.5E-124
Fibroblasts	H1F0	0.972847	0.271	3.7E-145
Fibroblasts	HSPE1	0.973609	0.632	4.5E-24
Fibroblasts	HNRNPDL	0.973892	0.576	1.47E-15
Fibroblasts	RPS2	0.974035	0.775	2.1E-21
Fibroblasts	CLMP	0.976858	0.305	0
Fibroblasts	RNASEK	0.977763	0.277	4.37E-34
Fibroblasts	SEC62	0.978073	0.721	1.9E-18
Fibroblasts	HCLS1	0.978945	0.833	3.97E-53
Fibroblasts	NID1	0.979095	0.314	0
Fibroblasts	SET	0.980861	0.552	7.2E-20
Fibroblasts	NFKBIZ	0.981442	0.481	2.98E-39
Fibroblasts	UBB	0.983352	0.771	4.97E-39
Fibroblasts	ANGPTL2	0.984944	0.279	0
Fibroblasts	PPDPF	0.985653	0.509	2.24E-19
Fibroblasts	RTL8C	0.992176	0.335	2.9E-123
Fibroblasts	GOLIM4	0.992986	0.31	2.33E-32
Fibroblasts	PEBP1	0.993132	0.517	1.33E-14
Fibroblasts	SRP14	0.996402	0.747	1.88E-37
Fibroblasts	ST5	0.998316	0.286	0
Fibroblasts	ATP5MC2	0.998805	0.727	1.16E-30
Fibroblasts	FARP1	0.999469	0.325	0
Fibroblasts	ANXA2	1.005095	0.576	1.74E-24
Fibroblasts	EPAS1	1.007781	0.355	6.4E-219

Fibroblasts	LHFPL6	1.010948	0.323	0
Fibroblasts	PCDH18	1.011197	0.299	0
Fibroblasts	SDC2	1.013978	0.344	0
Fibroblasts	HSBP1	1.014778	0.561	2.47E-33
Fibroblasts	SEC11A	1.017039	0.589	7.43E-24
Fibroblasts	CCDC71L	1.01931	0.271	1.1E-209
Fibroblasts	AXL	1.019713	0.353	0
Fibroblasts	CD59	1.020611	0.42	7.49E-37
Fibroblasts	EPHX1	1.021116	0.359	3E-133
Fibroblasts	FXYD1	1.023011	0.253	0
Fibroblasts	HSPA8	1.025124	0.647	5.95E-16
Fibroblasts	MRPS21	1.028286	0.53	1.17E-20
Fibroblasts	TM4SF1	1.028855	0.258	6.3E-159
Fibroblasts	CAV2	1.029565	0.31	2.4E-217
Fibroblasts	RHOA	1.029933	0.68	3.68E-29
Fibroblasts	EMID1	1.034839	0.34	0
Fibroblasts	TOMM7	1.035066	0.675	3.43E-24
Fibroblasts	PRKG1	1.036223	0.299	0
Fibroblasts	SLA	1.039919	0.29	0
Fibroblasts	DYNLL1	1.041114	0.669	1.63E-19
Fibroblasts	PKM	1.042754	0.509	6.25E-29
Fibroblasts	PLEKHA5	1.043186	0.353	0
Fibroblasts	BTF3	1.043791	0.725	4.23E-34
Fibroblasts	STAT2	1.045033	0.374	7.1E-32
Fibroblasts	PRSS23	1.047668	0.288	2.5E-256
Fibroblasts	POLR2L	1.048112	0.675	9.13E-29
Fibroblasts	CSRP1	1.053291	0.342	5.76E-26
Fibroblasts	MAPK10	1.053292	0.275	0
Fibroblasts	PPIA	1.055084	0.699	7.07E-34
Fibroblasts	SRRM2	1.059497	0.567	2.92E-23
Fibroblasts	REXO2	1.060527	0.481	1.8E-33
Fibroblasts	MORF4L1	1.062662	0.632	1.3E-30
Fibroblasts	VCL	1.067598	0.364	8E-235
Fibroblasts	RPS26	1.068269	0.825	5.94E-22
Fibroblasts	RBPMS	1.077636	0.379	0
Fibroblasts	ATP5PD	1.077671	0.545	7.79E-17
Fibroblasts	LDHA	1.08099	0.626	1.39E-22
Fibroblasts	TGFB1I1	1.081121	0.314	0
Fibroblasts	SGCB	1.083129	0.355	8.5E-190
Fibroblasts	EFEMP2	1.083842	0.361	0
Fibroblasts	ATOX1	1.087475	0.617	1.13E-31
Fibroblasts	PRKAR2B	1.087953	0.329	0
Fibroblasts	CTLA4	1.089564	0.658	1.92E-51
Fibroblasts	CAVIN1	1.103327	0.372	0
Fibroblasts	MYL12B	1.103954	0.682	5.11E-41
Fibroblasts	TUBB	1.104918	0.515	1.65E-53

Fibroblasts	GZMB	1.105328	0.377	0
Fibroblasts	SUMO2	1.109987	0.703	5.4E-39
Fibroblasts	ARID5B	1.114541	0.561	4.74E-25
Fibroblasts	C2	1.116081	0.392	1.4E-251
Fibroblasts	TRA2A	1.116852	0.435	7.89E-18
Fibroblasts	PFN1	1.120079	0.639	9.83E-44
Fibroblasts	LAMC1	1.120157	0.366	1.1E-154
Fibroblasts	B4GALT1	1.120189	0.351	4.69E-47
Fibroblasts	NDUFC1	1.125061	0.58	3.33E-24
Fibroblasts	PPP1R15A	1.125105	0.623	2.68E-31
Fibroblasts	EDIL3	1.126733	0.312	0
Fibroblasts	MIF	1.131346	0.444	3.07E-18
Fibroblasts	NDUFA4	1.137127	0.723	1.59E-28
Fibroblasts	C9orf3	1.139847	0.403	0
Fibroblasts	PALLD	1.139995	0.348	0
Fibroblasts	CALU	1.140923	0.487	9.97E-14
Fibroblasts	SOD2	1.148204	0.751	2.5E-145
Fibroblasts	DDR2	1.149174	0.34	2.4E-277
Fibroblasts	FHL1	1.152387	0.377	0
Fibroblasts	COMMD6	1.161878	0.639	1.11E-32
Fibroblasts	VMP1	1.166853	0.561	2E-27
Fibroblasts	TMEM14C	1.169239	0.567	6.66E-38
Fibroblasts	LOXL2	1.17147	0.333	0
Fibroblasts	TRPA1	1.175518	0.284	0
Fibroblasts	GSN	1.179627	0.494	3.1E-180
Fibroblasts	FBN1	1.180293	0.381	0
Fibroblasts	IL7R	1.183147	0.554	1.07E-51
Fibroblasts	SRGN	1.184511	0.299	0
Fibroblasts	SEM1	1.192207	0.669	3.49E-40
Fibroblasts	KRT10	1.198894	0.47	4.55E-29
Fibroblasts	PPIC	1.200494	0.357	0
Fibroblasts	AC020916.1	1.209464	0.403	1.53E-21
Fibroblasts	NDUFS5	1.214433	0.693	1.39E-39
Fibroblasts	MAP1LC3A	1.214721	0.338	1.5E-258
Fibroblasts	DNAJA1	1.214903	0.665	6.44E-38
Fibroblasts	MXRA8	1.224678	0.372	0
Fibroblasts	SERPINB6	1.226532	0.478	1.2E-159
Fibroblasts	C12orf57	1.227135	0.645	1.54E-42
Fibroblasts	MINOS1	1.227387	0.595	1.56E-41
Fibroblasts	CXCL12	1.231867	0.275	0
Fibroblasts	TSPAN4	1.248951	0.431	1.4E-241
Fibroblasts	CALM2	1.250558	0.693	1.48E-42
Fibroblasts	SPRY1	1.251953	0.32	8.7E-244
Fibroblasts	SNX3	1.259186	0.519	6.54E-43
Fibroblasts	THBS1	1.261321	0.381	8.3E-152
Fibroblasts	IFITM1	1.265521	0.431	1E-218

Fibroblasts	MXRA5	1.27412	0.361	0
Fibroblasts	HNRNPU	1.274582	0.63	3.88E-43
Fibroblasts	RGS1	1.282337	0.602	3.44E-50
Fibroblasts	BGN	1.286776	0.32	0
Fibroblasts	IFI27	1.288801	0.556	1.9E-298
Fibroblasts	EPB41L2	1.291516	0.407	4.1E-239
Fibroblasts	FERMT2	1.292251	0.398	0
Fibroblasts	CAVIN3	1.293971	0.359	0
Fibroblasts	NFIA	1.29765	0.361	0
Fibroblasts	PLAU	1.298022	0.366	2.7E-226
Fibroblasts	STAT3	1.298113	0.563	1.65E-53
Fibroblasts	ARGLU1	1.298297	0.552	1.98E-38
Fibroblasts	SOCS3	1.299972	0.565	1.86E-31
Fibroblasts	AHI1	1.300951	0.424	1.79E-49
Fibroblasts	PXDN	1.302941	0.368	0
Fibroblasts	GPR183	1.304032	0.39	0
Fibroblasts	FUS	1.308494	0.634	5.74E-50
Fibroblasts	SPARCL1	1.30856	0.318	6.3E-290
Fibroblasts	VCAN	1.309374	0.5	2.4E-297
Fibroblasts	RHOC	1.313785	0.522	3.8E-234
Fibroblasts	DST	1.326074	0.476	5.1E-206
Fibroblasts	ENTPD1	1.332347	0.4	0
Fibroblasts	ICOS	1.337947	0.392	0
Fibroblasts	EMP3	1.346933	0.682	3.97E-28
Fibroblasts	PKIG	1.354957	0.413	6.9E-210
Fibroblasts	RRAS	1.355134	0.439	1.2E-223
Fibroblasts	LPP	1.358493	0.478	1.2E-175
Fibroblasts	RPS4Y1	1.361421	0.465	4.67E-28
Fibroblasts	NR4A1	1.363762	0.515	4.94E-26
Fibroblasts	TLE4	1.364799	0.409	9.66E-41
Fibroblasts	F2R	1.367501	0.377	6.6E-166
Fibroblasts	QSOX1	1.378134	0.385	3.5E-168
Fibroblasts	TCEAL9	1.398344	0.481	7.8E-185
Fibroblasts	CSRP2	1.401659	0.26	6.5E-240
Fibroblasts	TCF4	1.416638	0.552	2.68E-46
Fibroblasts	TCEAL4	1.420687	0.494	1.6E-151
Fibroblasts	MT-CO1	1.429412	0.987	2.3E-130
Fibroblasts	ADAMTS1	1.451175	0.251	6E-268
Fibroblasts	TIMP2	1.451308	0.498	1.4E-228
Fibroblasts	IL6ST	1.467731	0.517	9.5E-121
Fibroblasts	MGST3	1.469614	0.626	1.58E-38
Fibroblasts	FTH1	1.476361	0.942	9.5E-128
Fibroblasts	SLC38A2	1.481605	0.578	4.75E-28
Fibroblasts	DNAJB1	1.484045	0.517	5.63E-36
Fibroblasts	NFIC	1.508348	0.504	9.6E-286
Fibroblasts	CYTIP	1.51602	0.323	0

Fibroblasts	YBX3	1.522132	0.613	3.1E-189
Fibroblasts	BIRC3	1.532984	0.405	0
Fibroblasts	RHOH	1.550955	0.253	0
Fibroblasts	IGFBP6	1.554853	0.366	0
Fibroblasts	ID1	1.555852	0.32	2.5E-151
Fibroblasts	FSTL1	1.557351	0.463	0
Fibroblasts	IRF1	1.57424	0.604	2E-28
Fibroblasts	IL1R1	1.576489	0.476	0
Fibroblasts	PPP1R14A	1.578558	0.316	0
Fibroblasts	ZFP36L2	1.596661	0.634	3.8E-37
Fibroblasts	ADH1B	1.602288	0.262	0
Fibroblasts	PMP22	1.604111	0.522	0
Fibroblasts	PDLIM1	1.61099	0.55	3.5E-132
Fibroblasts	HNRNPH1	1.612325	0.71	4.05E-48
Fibroblasts	RAB13	1.624076	0.552	6.8E-164
Fibroblasts	GNG11	1.624614	0.483	0
Fibroblasts	PTMS	1.625285	0.558	3.8E-268
Fibroblasts	COX7A1	1.626965	0.502	0
Fibroblasts	CRISPLD2	1.627768	0.444	0
Fibroblasts	PDPN	1.635798	0.448	0
Fibroblasts	LRP1	1.638448	0.494	0
Fibroblasts	COL5A1	1.641264	0.442	0
Fibroblasts	CNN3	1.649284	0.548	0
Fibroblasts	PHLDA2	1.649435	0.271	7.4E-154
Fibroblasts	COL12A1	1.651966	0.364	0
Fibroblasts	CD151	1.652883	0.578	2.8E-176
Fibroblasts	GEM	1.661326	0.374	0
Fibroblasts	TGFBI	1.66677	0.47	5.2E-255
Fibroblasts	COL4A1	1.671227	0.374	0
Fibroblasts	MALAT1	1.675923	0.991	6.5E-122
Fibroblasts	HES1	1.67914	0.348	3.2E-173
Fibroblasts	NBL1	1.688996	0.511	0
Fibroblasts	FOSB	1.690104	0.688	2E-45
Fibroblasts	TSC22D1	1.693642	0.448	2.2E-192
Fibroblasts	COL14A1	1.699477	0.396	0
Fibroblasts	PROCR	1.713539	0.485	0
Fibroblasts	TNC	1.715702	0.299	0
Fibroblasts	NR2F1	1.731022	0.411	0
Fibroblasts	GADD45B	1.740072	0.465	9E-47
Fibroblasts	SPON2	1.748085	0.433	0
Fibroblasts	AEBP1	1.748745	0.515	0
Fibroblasts	PDGFRB	1.764788	0.435	0
Fibroblasts	NUPR1	1.766935	0.578	0
Fibroblasts	BMP4	1.768002	0.379	0
Fibroblasts	COL18A1	1.769897	0.459	0
Fibroblasts	GBP5	1.770665	0.463	0

Fibroblasts	RBP1	1.772089	0.424	0
Fibroblasts	LGALS3	1.776731	0.788	1.2E-111
Fibroblasts	PHLDA1	1.779148	0.506	1.1E-286
Fibroblasts	KLF2	1.7806	0.442	2.09E-35
Fibroblasts	C3	1.781715	0.292	0
Fibroblasts	COL5A2	1.818512	0.483	0
Fibroblasts	FGF7	1.858168	0.372	0
Fibroblasts	ZFP36L1	1.860813	0.792	9.6E-162
Fibroblasts	TCF21	1.872847	0.452	0
Fibroblasts	CD81	1.873662	0.703	4E-138
Fibroblasts	CCDC80	1.881624	0.381	0
Fibroblasts	CFH	1.897104	0.509	0
Fibroblasts	VIM	1.912003	0.877	4.2E-123
Fibroblasts	ADIRF	1.915869	0.327	0
Fibroblasts	CYGB	1.918425	0.459	0
Fibroblasts	IER3	1.925662	0.554	1.1E-121
Fibroblasts	PLK2	1.938035	0.372	2.9E-288
Fibroblasts	SOD3	1.960594	0.424	0
Fibroblasts	RND3	1.965842	0.468	0
Fibroblasts	ITGB1	2.162162	0.682	9.2E-170
Fibroblasts	S100A4	2.253585	0.79	6.3E-139
Fibroblasts	S100A10	2.275183	0.716	5E-112
Fibroblasts	CEBPB	2.28543	0.613	3.5E-193
Fibroblasts	ATF3	2.307155	0.55	6.2E-115
Fibroblasts	TUBA1A	2.309141	0.673	2.5E-278
Fibroblasts	CXCL1	2.440989	0.366	7.9E-250
Fibroblasts	HSPB1	2.496844	0.688	3.2E-228
Fibroblasts	CST3	2.507892	0.779	8E-294
Fibroblasts	LGALS1	2.590995	0.835	6.5E-117
Fibroblasts	JUN	2.666737	0.866	2.4E-134
Fibroblasts	CTSC	2.666852	0.742	1.1E-121
Fibroblasts	FOS	2.819822	0.907	2.1E-163
Fibroblasts	S100A6	2.832981	0.939	2.6E-201
Fibroblasts	TIMP1	2.974534	0.87	1.3E-231
Fibroblasts	ACTA2	2.978662	0.266	1.3E-261
Fibroblasts	ID3	3.269034	0.658	6.8E-244
Fibroblasts	CXCL14	5.741794	0.693	0
